# Elevated exopolysaccharide levels in *Pseudomonas aeruginosa* flagellar mutants have implications for biofilm growth and chronic infections

**DOI:** 10.1371/journal.pgen.1008848

**Published:** 2020-06-12

**Authors:** Joe J. Harrison, Henrik Almblad, Yasuhiko Irie, Daniel J. Wolter, Heather C. Eggleston, Trevor E. Randall, Jacob O. Kitzman, Bethany Stackhouse, Julia C. Emerson, Sharon Mcnamara, Tyler J. Larsen, Jay Shendure, Lucas R. Hoffman, Daniel J. Wozniak, Matthew R. Parsek

**Affiliations:** 1 Department of Biological Sciences, University of Calgary, University Drive NW, Calgary, AB, Canada; 2 Department of Microbiology, University of Washington, Seattle, Washington, United States of America; 3 Department of Pediatrics, University of Washington, Seattle, Washington, United States of America; 4 Department of Microbial Infection and Immunity, Department of Microbiology, Center for Microbial Interface Biology, The Ohio State University, Columbus, Ohio, United States of America; 5 Department of Genome Sciences, University of Washington, Seattle, Washington, United States of America; 6 Center for Clinical and Translational Research, Seattle Children’s Hospital, Seattle, Washington, United States of America; The University of Texas Health Science Center at Houston, UNITED STATES

## Abstract

*Pseudomonas aeruginosa* colonizes the airways of cystic fibrosis (CF) patients, causing infections that can last for decades. During the course of these infections, *P*. *aeruginosa* undergoes a number of genetic adaptations. One such adaptation is the loss of swimming motility functions. Another involves the formation of the rugose small colony variant (RSCV) phenotype, which is characterized by overproduction of the exopolysaccharides Pel and Psl. Here, we provide evidence that the two adaptations are linked. Using random transposon mutagenesis, we discovered that flagellar mutations are linked to the RSCV phenotype. We found that flagellar mutants overexpressed Pel and Psl in a surface-contact dependent manner. Genetic analyses revealed that flagellar mutants were selected for at high frequencies in biofilms, and that Pel and Psl expression provided the primary fitness benefit in this environment. Suppressor mutagenesis of flagellar RSCVs indicated that Psl overexpression required the *mot* genes, suggesting that the flagellum stator proteins function in a surface-dependent regulatory pathway for exopolysaccharide biosynthesis. Finally, we identified flagellar mutant RSCVs among CF isolates. The CF environment has long been known to select for flagellar mutants, with the classic interpretation being that the fitness benefit gained relates to an impairment of the host immune system to target a bacterium lacking a flagellum. Our new findings lead us to propose that exopolysaccharide production is a key gain-of-function phenotype that offers a new way to interpret the fitness benefits of these mutations.

## Author summary

Microbiologists have known for decades that *Pseudomonas aeruginosa* mutates during chronic respiratory infection of cystic fibrosis (CF) patients. One of the most reported functions lost during these infections is flagellar motility. A long-standing interpretation of this observation is that the flagellum is disadvantageous for the bacterium in the CF environment. We report the surprising finding that mutation of a wide range of flagellar genes results in the overproduction of the biofilm matrix polysaccharides Psl and Pel. We propose, therefore, that flagellar mutations represent a gain-of-function that would help the bacterium to form biofilms and persist in the CF airways.

## Introduction

One of the most important CF pathogens is *Pseudomonas aeruginosa* [1]. During chronic infection, it predictably acquires a number of adaptive mutations that are believed to aid in long-term persistence [2–4]. These adaptations include alginate overproduction [5], amino acid auxotrophy [6], loss of lipopolysaccharide O-antigens [7] and loss of flagellar motility [8]. Although the fitness benefits that these different mutations confer is uncertain, several seem intuitive. For example, amino acid auxotrophy may relieve the metabolic burden of amino acid biosynthesis in the amino acid rich environment of CF airway secretions. Another is the loss of motility functions, which is thought to assist in the evasion of the host immune system by eliminating production of a key antigen and pathogen-associated molecular pattern, flagellin.

**Fig 1:**
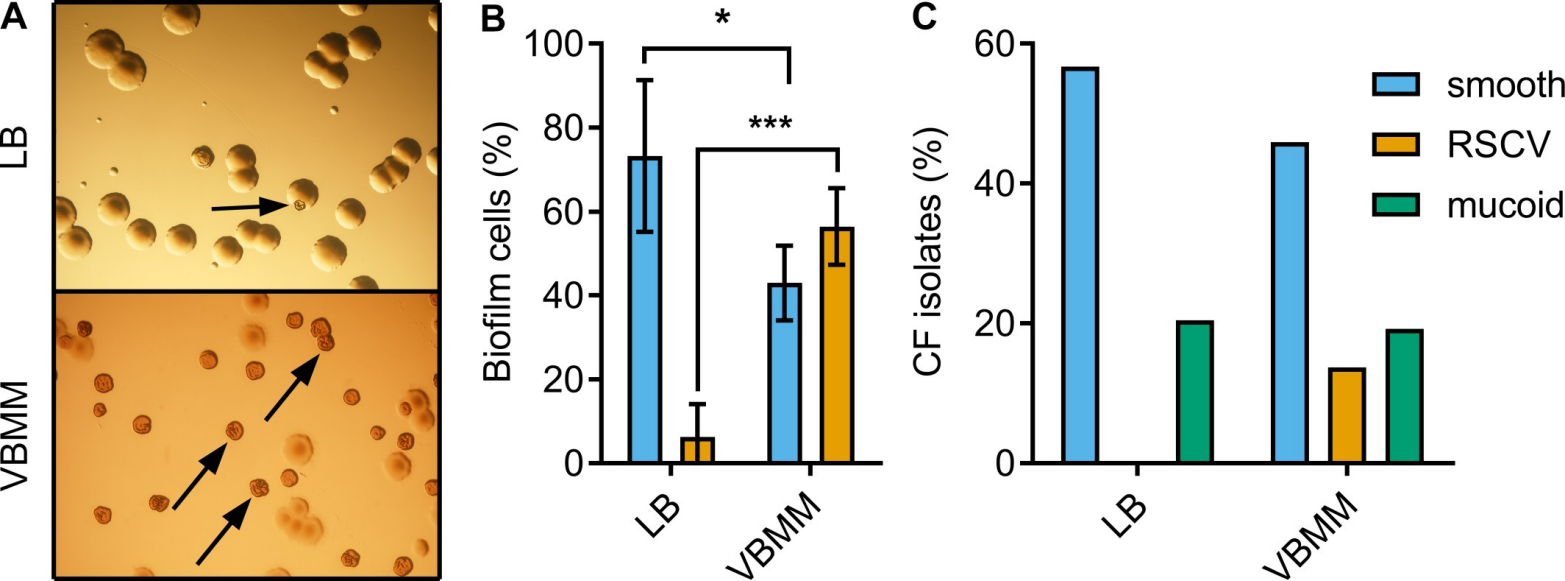
RSCVs are abundant in *P*. *aeruginosa* biofilms and among CF isolates. (A) An aliquot of cells recovered from a single drip-flow reactor biofilm was plated onto LB or VBMM agar. The arrows indicate representative RSCVs from each sample. Each panel represents an area that is approximately 22.9 mm × 16.0 mm. (B) Proportion of RSCV and ancestral smooth colony morphotypes on LB and VBMM agar. Each bar indicates the mean and standard deviation for 6 independent drip-flow reactors. *P<0.05 and ***P ≤ 0.001 colony counts on LB vs. VBMM agar with Student’s t-test. (C) A total of 416 *P*. *aeruginosa* isolates acquired from a study of CF respiratory epidemiology were streaked on LB or VBMM-based agar (see S1 Text) and scored for colony morphology. The percentage of isolates with the indicated colony morphologies is illustrated.

Several similar genetic adaptations are observed in laboratory biofilms. This is potentially important because CF airway infections are thought to have biofilm etiology [9, 10]. One *P*. *aeruginosa* phenotype that is selected for in laboratory biofilms is the **r**ugose **s**mall **c**olony **v**ariant (RSCV) [11, 12] (Fig 1A). These variants have also been isolated from CF sputum [12–15]. RSCVs make profuse amounts of the extracellular polysaccharides (EPS) Pel and Psl as well as the extracellular adhesin CdrA [16, 17]. Expression of Pel and Psl is associated with tolerance to some clinically-relevant antibiotics [18, 19], and it safeguards *P*. *aeruginosa* against complement-mediated opsonization and neutrophil phagocytosis [20]. Pel, Psl and CdrA are also known to play a role in biofilm aggregate formation [17, 18, 21], which is associated with protection from antimicrobials [22–24] and host defenses [16, 25]. It is not surprising, therefore, that prior reports have associated the appearance of RSCVs in CF sputum to poor patient outcomes [26, 27].

Expression of the RSCV morphotype is linked to elevated levels of the second messenger cyclic diguanylate (c-di-GMP) [28–32]. Several loci have been linked to the RSCV phenotype, including the *wsp* operon [29], the *tpb* (also denoted *aws* or *yfi*) loci [28, 33, 34], *amrZ* [35, 36], *dsbA* [37], genes of the *rsm* signalling pathway [21, 38] and *pvrR* [30]. Previous work has estimated that RSCVs represent approximately 5% of laboratory grown biofilm populations and that *wsp* mutations account for a majority of these [16]. RSCVs harbouring mutations in the *wsp* and *yfi* loci have also been observed among CF isolates [2, 39].

In this study, we found that the frequency of RSCVs in laboratory biofilms and CF isolates has been grossly underestimated. Using transposon mutagenesis, we sought to identify novel genetic elements linked to the phenotype. We found 22 genes in our screen that we grouped into four classes. Perhaps the most surprising class were mutations in genes encoding flagellum structure and function. Unlike the other classes of RSCVs, flagellar mutants overexpressed Pel and Psl in a surface-contact-dependent fashion. We also found that flagellar mutations are selected for at high frequency in laboratory biofilms, and that increased Pel and Psl expression provides the primary fitness benefit. Suppressor mutagenesis revealed that Psl overexpression by flagellar mutants requires the *mot* genes as well as components of the Pil-Chp surface-sensing system. Finally, we show that flagellar mutant RSCVs are also found among CF clinical isolates. We propose that gain-of-function phenotypes (Pel and Psl overexpression) offer a new interpretation of the benefits of flagellar mutations in isolates from laboratory biofilms and CF infections.

## Results and discussion

### *P*. *aeruginosa* RSCVs are abundant in laboratory biofilms and CF isolates

*P*. *aeruginosa* biofilms grown *in vitro* rapidly undergo mutation and selection [11]. A manifestation of this is the appearance of diverse colony morphotypes when biofilm bacteria are plated onto solid growth media. Using a growth medium optimized for RSCV detection (Vogel-Bonner minimal medium supplemented with the dyes Congo Red and Coomasie brilliant blue), we found that RSCVs are observed ~9-fold more frequently than on traditional lysogeny broth (LB) agar (Fig 1A and 1B). Many isolates displaying the RSCV phenotype on VBMM had a wild type smooth colony morphotype when re-streaked on LB agar.

A key question for these isolates is whether RSCV-linked traits (elevated Pel and Psl production) are only expressed on VBMM-based agar compared to LB. To test this, Psl and PelC expression were measured for strains that exhibited the RSCV phenotype on VBMM but were smooth when grown on LB. We observed that all these strains still overexpressed PelC and/or Psl on both VBMM and LB (S1 Fig). These data indicate that elevated EPS expression is a feature of these strains regardless of the growth medium or colony morphotype.

We next asked if RSCVs are more common than previously thought among CF sputum isolates (Fig 1C). We examined 416 *P*. *aeruginosa* isolates cultured from 46 pediatric CF patients enrolled in a two-year study of respiratory bacterial epidemiology at Seattle Children's Hospital [40]. Only a single isolate had an RSCV phenotype on LB agar. By contrast, 56 (13.5%, Fig 1C) of these isolates (from 40% of the patients) displayed the RSCV phenotype on VBMM-based agar medium. By comparison, perhaps the best-known CF-related *P*. *aeruginosa* adaptive colony morphology, mucoidy [41], was observed in 80 (19.2%, Fig 1C) of the isolates (from 49% of the patients).

### Identification of genes that are linked to the RSCV phenotype

Work by Starkey and colleagues [16] indicated that *wspF* mutations account for 70% of the RSCVs that are isolated on LB agar from biofilms. We randomly isolated 25 RSCVs on VBMM agar from biofilms (i.e. 5 RSCVs from each of 5 different drip-flow biofilm reactors in order to increase the likelihood that these RSCVs were non-isogenic) and attempted to complement these mutants with a wild type copy of the *wspF* gene; however, in only a single instance did this restore the wild type colony morphology of these biofilm isolates. Analogously, transformation of 10 RSCVs (from 10 different patients) recovered on VBMM-based agar from CF sputum with a plasmid bearing *wspF* restored smooth colony morphology in only 3 of 10 isolates. These data suggest that the majority of RSCVs isolated from biofilms and from CF sputum may be *wsp*-independent.

To identify *wsp*-independent genes linked to the RSCV phenotype, we carried out near-saturation transposon mutagenesis of *P*. *aeruginosa* PAO1 harboring an engineered deletion of *wspR*, the diguanylate cyclase of the *wsp* system [29]. This allowed us to eliminate the isolation of *wsp*-related mutations that might confer the RSCV phenotype. Here we used the transposon miniTn5Pro, which carries the repressor *araC* as well as an outward-facing, arabinose-inducible promoter [42]. Approximately 35,000 miniTn5Pro mutants were scored for colony morphology on selective VBMM agar with and without arabinose. This random gene-disruption-gene-activation strategy produced 57 genetically distinct RSCVs in which the transposon was mapped to 22 different genes from 17 different operons (Table 1). Collectively, the identified genetic elements could be placed into four groups based on known and predicted functions: 1) the regulator of secondary metabolism (*rsm*) signalling pathway, 2) the periplasmic thiol-disulfide interchange protein (*dsb*A), 3) flagellum biosynthesis, and 4) diguanylate cyclases and phosphodiesterases that synthesize and degrade c-di-GMP, respectively. We also placed oligoribonuclease (*orn*) in this last category. The *orn* gene encodes a 3ʹ→5ʹ exoribonuclease that degrades pGpG, an intermediate product produced by EAL-domain phosphodiesterases in the two-step pathway for c-di-GMP degradation [43, 44]. Only a single mutant displayed an arabinose inducible RSCV phenotype, and in this case the transposon insertion point was mapped upstream of the diguanylate cyclase *siaD*.

**Table 1:** *P*. *aeruginosa* PAO1 Δ*wspR* transposon mutants with an RSCV phenotype.

Insertion site^1^	# insertions	Function of disrupted gene^2^	Operonic structure^2^ (class^3^)
**Regulator of secondary metabolism (*rsm*) signalling pathway**			
*retS*	3	regulator of exopolysaccharide and type III secretion, sensor histidine kinase	monocistronic
**Redox homeostasis and protein folding**			
*dsbA*	2	thiol-disulfide interchange protein	*dsbA-PA5488-PA5487*
**Flagellum biosynthesis**			
*flhA*	1	flagellum export component, membrane target for soluble export complex	monocistronic (II)
*flhB*	1	flagellum export component, substrate specificity switch, target for soluble export complex	*fliLMNOPQRflhB* (II)
*flhF*	2	predicted exporter with FHIPEP family motif	*flhF-fleN* (II)
*fliF*	5	flagellum M-ring, outer membrane protein	*fliEFGHIJ* (II)
*fliH*	1	putative flagellum TTSS protein	*fliEFGHIJ* (II)
*fliI*	1	flagellum specific ATP synthase	*fliEFGHIJ* (II)
*fliM*	3	flagellum motor switch protein	*fliLMNOPQRflhB* (II)
*fliO*	3	flagellum export component	*fliLMNOPQRflhB* (II)
*fliD*	2	flagellum capping protein	*fliDSSʹfleP* (II)
*flgI*	1	flagellum basal body P-ring protein	*flgFGHIJKL* (III)
*flgJ*	2	muraminadase	*flgFGHIJKL* (III)
*flgK*	3	flagellum hook-associated protein	*flgFGHIJKL* (III)
*flgL*	1	flagellum hook-associated protein	*flgFGHIJKL* (III)
*fliC*	7	flagellin, type B	*fliCfleL* (IV)
*flgN*	2	chaperone protein, initiation of filament assembly	*flgMNZ* (IV)
**Diguanylate cyclases and phosphodiesterases**			
*orn*	2	oligoribonuclease	monocistronic
*siaB*^1^^,^^4^	1	hypothetical protein, unknown function, predicted serine phosphatase domain	*siaABCD*
*PA1850*^1^	2	*araC* like transcription factor with an amidase domain, predicted DJ-1/ThiJ/PfpI family protein	*PA1850-PA1849-PA1848*
*dipA*	5	GAF-PAS-ASNEF-EAL protein	*dipA-msrA*
*PA5295*	4	COG5001-GGDEF-EAL domain protein	monocistronic

^1^The outwards facing *araC*-*P*_*BAD*_ of miniTn5Pro was located upstream of *siaD* and *PA1851*, which are putative diguanylate cyclases.

^2^Predicted annotations and operonic structures for flagellar genes were taken from Dasgupta and colleagues [45]. All other annotations and operonic structures were retrieved from the Pseudomonas Genome Database [82] on April 5, 2020.

^3^Class as defined by the established four-tier flagellar gene transcriptional hierarchy in *P*. *aeruginosa* by Dasgupta and colleagues [45].

^4^This transposon mutant was arabinose responsive.

**Fig 2:**
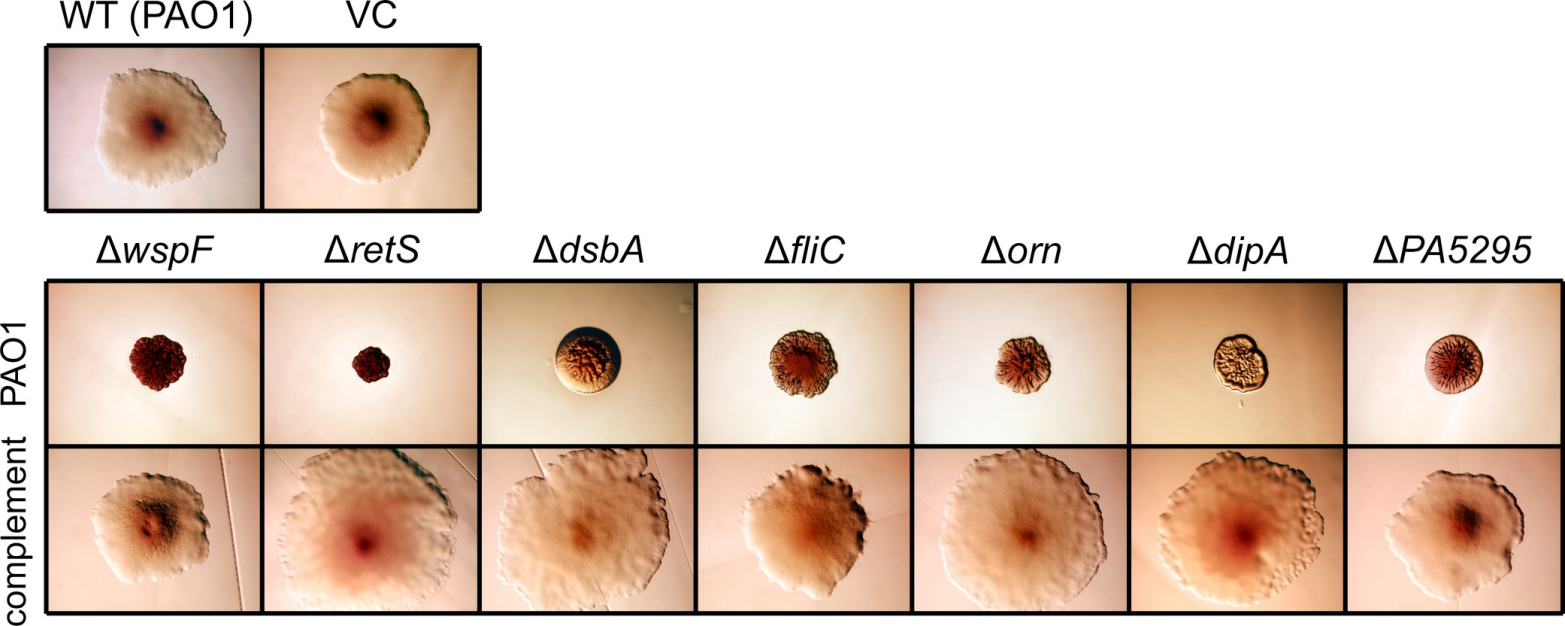
Multiple genes are linked to the RSCV phenotype. Precisely defined in-frame deletion mutations recreated the RSCV phenotype of transposon mutants. Mutations were complemented by expressing the deleted gene from its native promoter *in trans*. Here, this was done using a miniTn7 vector to insert a single copy of the deleted gene at the *glmS* site of the *P*. *aeruginosa* chromosome. Insertion of an empty miniTn7 vector into chromosome did not affect colony morphology. In all panels, bacteria were cultured and photographed on VBMM agar containing Congo red and brilliant blue R (see Material and Methods). Each panel represents an area that is approximately 5.0 mm × 3.5 mm. WT, wild type; VC, vector control.

A limitation of transposon mutagenesis is that it is difficult to predict whether transposon insertions cause polar effects that disrupt the expression of adjacent genes in operons. Therefore, unmarked deletion alleles were constructed for genes chosen to represent each of the four groups of RSCV-linked mutations. As expected, introducing these deletion alleles into the wild type PAO1 (Fig 2) and Δ*wspR* strains (S2A Fig) duplicated the RSCV phenotype of transposon mutants. Each of these mutants had smooth, and in some instances small, colony morphology on LB agar (S2B Fig). Each mutant strain was then complemented using miniTn7 to insert a single copy of the deleted gene and its native promoter elsewhere on the chromosome (Fig 2). Additionally, precise, in-frame deletion mutations were constructed in ten flagellar genes. Genes were chosen to represent operons from each of the known tiers (Table 1) of the four-tier flagellar gene regulation hierarchy that orders flagellum assembly in *P*. *aeruginosa* [45]. Our results show that some but not all disruptions in flagellar operons may result in rugose colony morphology on VBMM (S3 Fig). Moreover, with the exception of Δ*fleQ* (which is at the first tier of flagellar gene regulation), we noted that RSCV-linked mutations can occur in operons at every tier of the flagellar gene regulation hierarchy. Taken together, these data suggest that the assembly status of the *P*. *aeruginosa* flagellum regulates EPS production, which is similar to observations previously reported for other bacterial species [46, 47].

### Flagellar mutants overproduce EPS in a surface contact-dependent fashion

**Fig 3:**
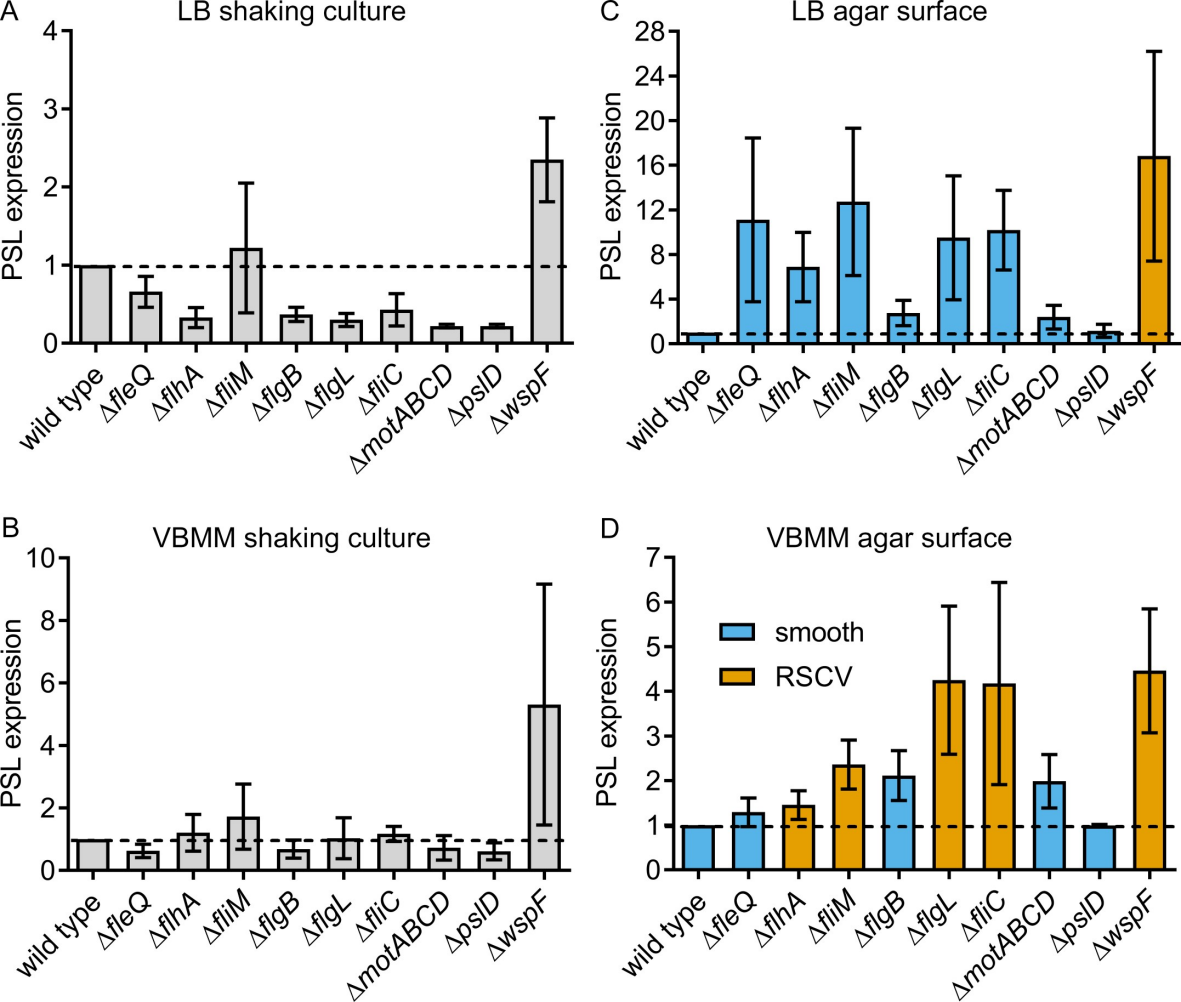
Overexpression of Psl by flagellar mutants is surface-contact dependent. Semi-quantitative dot blots for the Psl polysaccharide from flagellar mutants grown (A and B) in shaken LB or VBMM cultures, respectively, or (C and D) on the surface of LB or VBMM agar, respectively. Each bar indicates the mean and standard deviation for 1 to 3 technical replicates from each of 3 independent biological replicates.

Transcriptional profiling has identified a role for the master flagellar regulator FleQ in repressing the *pel* and *psl* operons [45]. However, there is little evidence that other flagellar mutations increase the expression of EPS genes. Here, we assessed molecular markers of EPS production for four flagellar mutants that expressed the RSCV phenotype on VBMM (Δ*flhA*, Δ*fliM*, and Δ*flgL* and Δ*fliC*) and for three that did not (Δ*fleQ*, Δ*flgB* and Δ*motABCD*). When grown in shaking liquid culture, none of these mutants displayed increased expression of Psl (Fig 3A and 3B). Moreover, apart from Δ*fleQ*, none showed overexpression of PelC (S4 Fig). By contrast, nearly all these mutants showed elevated Psl when grown on solid medium (Fig 3C and 3D). Except for the Δ*motABCD* strain, nearly all these mutants also overexpressed PelC when grown on VBMM agar (S4 Fig). These data suggest that surface contact might be a stimulus for flagellar mutants to express biofilm matrix polysaccharides. These observations are consistent with reports that the flagellum has a role in mechanosensation that is similar to other bacteria [48], including *V*. *cholerae* [49], *Vibrio paraheamolyticus* [50], *Caulobacter crescentus* [51], *Proteus mirabilis* [52] and *Bacillus subtilis* [53].

### Suppressor mutagenesis reveals a role for the flagellum stator and the Pil-Chp surface-sensing system in regulating EPS production in flagellar mutants

To reveal the regulatory pathway that links flagellar mutations to EPS overexpression, we carried out transposon mutagenesis of the Δ*fliC* strain. We again utilized minTn5Pro to enable random gene disruption and activation, but this time sought Δ*fliC* transconjugates that exhibited a smooth colony morphology. Approximately 21,000 Δ*fliC* miniTn5Pro mutants were evaluated on VBMM agar with or without arabinose, and this analysis yielded 115 genetically distinct mutants in which the transposon was mapped to 70 genes. A subset of 58 candidate second-site suppressor mutations, none of which were arabinose-responsive, mapped to only 26 genes in 17 operons (Table 2).

**Table 2:** Transposon suppressor mutations of the *P*. *aeruginosa* PAO1 Δ*fliC* RSCV phenotype.

Insertion site	# insertions	Function of disrupted gene^1^	Operonic structure^1^ (class^2^)
**Regulator of secondary metabolism (*rsm*) signalling pathway**			
*gacS*	1	global activator of cyanide synthesis, sensor histidine kinase, signal transduction	*gacSldhA-PA0926-PA0925*
**Flagellum biosynthesis**			
*fleQ*	5	transcriptional regulator	monocistronic (I)
*fleN*	4	flagellar synthesis regulator	*flhFfleN* (II)
*motA*	4	flagellum stator protein, exerts torque against motor switch	*motAB*
*motB*	5	flagellum stator protein, converts proton energy into torque	*motAB*
*motC*	1	flagellum stator protein, exerts torque against motor switch	*PA1458-PA1459-motCD-PA1462*
*PA1462*	1	unknown function	*PA1458-PA1459-motCD-PA1462*
**C-di-GMP signal transduction**			
*sadB*	6	unknown function	monocistronic
*sadC*	2	GDDEF domain protein	*sadC-PA4331-PA4330*
*siaA*	3	hypothetical protein, predicted HAMP domain	*siaABCD*
*siaB*	1	hypothetical protein	*siaABCD*
*siaD*	1	GGDEF domain protein, diguanylate cyclase	*siaABCD*
**Type IV pilus**			
*fimX*	1	type four pilus biosynthesis, GGDEF-EAL domain protein	*fimWfimX*
*pilB*	2	type four fimbrial biogenesis protein	monocistronic
*pilC*	1	type four fimbrial biogenesis protein	*pilCDcoaE-PA4530*
*pill*	1	component of chemotactic signal transduction system, response regulator	*pilGHI*
*pilM*	2	type four fimbrial biogenesis assembly protein, ATPase	*pilMNOPQ*
*pilO*	1	type four pilus assembly, O-glycosyltransferase	*pilMNOPQ*
*pilQ*	1	type four fimbrial biogenesis outer membrane protein precursor	*pilMNOPQ*
*pilR*	1	two-component response regulator	*pilSR*
*pilW*	2	type four fimbrial biogenesis protein	*fimUpilVWXY1Y2E*
*pilY1*	4	type four fimbrial biogenesis protein, tip associated adhesin	*fimUpilVWXY1Y2E*
**Unknown function**			
*PA1766*	2	hypothetical protein, predicted ATP-grasp domain	*PA1768-PA1767-PA1766*
*PA1767*	4	hypothetical protein, predicted cytoplasmic membrane protein	*PA1768-PA1767-PA1766*
*PA1768*	1	hypothetical protein, predicted type I export signal	*PA1768-PA1767-PA1766*
*PA1769*	1	hypothetical protein	monocistronic

^1^Annotations and predicted operonic structures for flagellar genes were taken from Dasgupta and colleagues [45]. All other annotations and predicted operonic structures were retrieved from the Pseudomonas Genome Database [82] on April 5, 2020.

^2^Class as defined by the established four-tier flagellar gene transcriptional hierarchy in *P*. *aeruginosa* by Dasgupta and colleagues [45].

**Fig 4:**
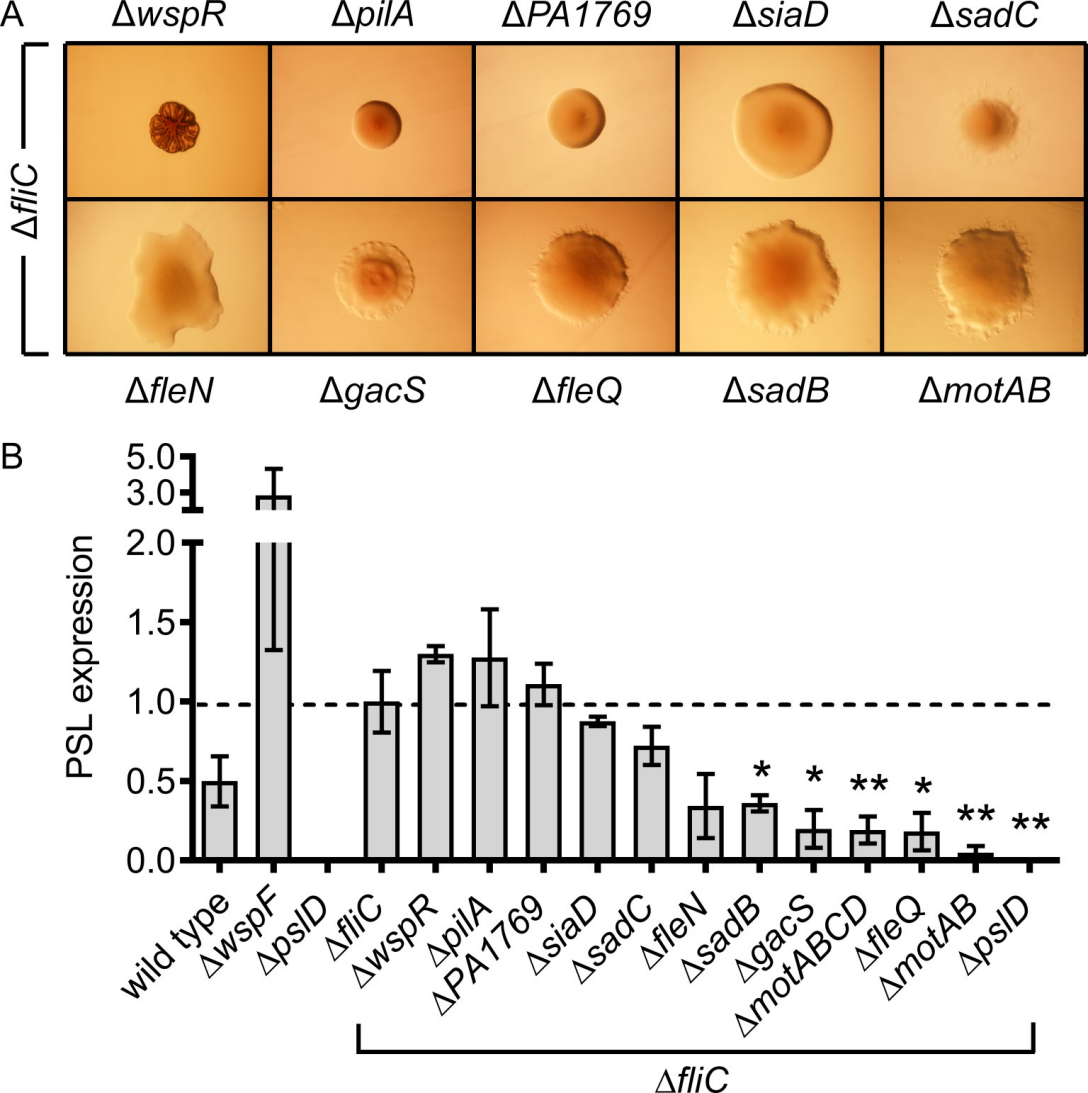
Suppressor mutagenesis suggests a role for the flagellum stator proteins and the Pil-Chp system in EPS biosynthesis by flagellar mutant RSCVs. (A) Precisely defined in-frame deletion mutations were engineered into the chromosome of the PAO1 Δ*fliC* strain. In all panels, bacteria were cultured and photographed on VBMM agar containing Congo red and brilliant blue R (see Material and Methods). Each panel represents an area that is approximately 5.0 × 3.5 mm. (B) Semi-quantitative anti-Psl ELISA assays of the Δ*fliC* strain bearing second-site suppressor mutations. Each bar represents the mean and standard error of 3 to 6 independent biological replicates. *P<0.05 and **P ≤ 0.005 vs. Δ*fliC* with Student’s t-test.

To further investigate these findings, in-frame deletion mutations were constructed in genes representing key suppressor mutations: *gacS*, *fleQ*, *fleN*, *motAB*, *motABCD*, *sadB*, *sadC*, *siaD*, *pilA*, and *PA1769*. Introducing these deletions into the Δ*fliC* background in all cases abolished the RSCV-phenotype on VBMM (Fig 4A). To further distinguish suppression of EPS expression from changes in colony morphology, we used an enzyme-linked immunosorbent assay (ELISA) to quantify the amount of Psl produced by each of these double, triple and quintuple mutants. Mutations in *gacS*, *fleQ*, *sadB*, *motAB* and *motABCD* signifcantly reduced Psl production by the Δ*fliC* strain (Fig 4B). Together, *motAB* or *motCD* encode a stator complex that provides flagellar torque [54, 55]. Here, we conclude that EPS overexpression by flagellar mutants involves a regulatory pathway that not only requires SadB [56–58], but also involves the flagellum stator proteins. These observations are consistent with a recent report from Baker and colleagues [59] in which the MotCD stator was observed to interact with the diguanylate cyclase SadC to stimulate c-di-GMP production under conditions not permissive to motility. SadC was also identified in our suppressor screen. In addition, we found a number of type IV pilus genes linked to the Pil-Chp surface sensing system, including four separate insertions in *pilY1* (Table 2), which encodes a pilus tip protein that has a putative mechanosensory domain [60]. We also found four separate insertions in the *pilMONPQ* operon (Table 2), which encodes the pilus alignment complex that is thought to transduce stimuli from PilY1 [61]. Because a Δ*pilA* Δ*fliC* double mutant did not have decreased levels of Psl production (Fig 4), one interpretation of these data is that type IV pilus-dependent motility might be important for the RSCV phenotype of flagellar mutants; however, another possibility is that the stator-dependent pathway of surface-sensing requires some parts of the Pil-Chp surface-sensing system to stimulate the RSCV phenotype on agar surfaces. While a complete mechanism is not yet evident, a teleological explanation is that altered function of flagellum motor components engages a signal transduction pathway that leads to biofilm formation. These observations might be informative to our understanding of the early stages of biofilm formation.

These observations are not without precedent: flagellin (*flaA*) mutants of *Vibrio cholerae* O139 also display a rugose colony morphology [47]. In *V*. *cholerae*, this gain-of-function phenotype depends on the *Vibrio* polysaccharide (*vps*) genes, the master regulator of the *vps* gene cluster (VpsR) and the sodium-driven flagellar motor (MotX) [62]. Work by Wu and colleagues [46] has also identified a stator-dependent signal transduction process that regulates *V*. *cholerae* EPS gene expression by modulating intracellular c-di-GMP. This flagellum-dependent biofilm response (FDBR) depends on the activities of at least three different diguanylate cyclases [46]. Under certain conditions and similar to *P*. *aeruginosa* [63, 64], *V*. *cholerae* O139 flagellar mutants grown on glass coverslips can form thick biofilm monolayers or microcolonies with architectures that are distinct from wild type [47]. Additionally, EPS-producing *V*. *cholerae* has an advantage in biofilm competition against isogenic, EPS-deficient strains; however, a cost of EPS-production is an impaired ability to disperse to new locations [65].

### Flagellar mutants predictably and reproducibly evolve in laboratory biofilms

One context in which EPS-overproducing flagellar mutants might have an advantage is in biofilms. EPS-mediated adhesion may allow bacteria to better occupy space on the substratum [66] and polymer production may provide better access to oxygen and nutrients as biofilms grow [67]. To test the hypothesis that flagellar mutants might account for RSCVs isolated from *in vitro* grown biofilms (Fig 1), we began by inoculating five independent reactors with a founding population of wild type PAO1 taken from a single, shaken overnight culture. After 5 d, a random sample of RSCVs was isolated on VBMM agar from each reactor. Genome sequencing revealed that RSCV-linked mutations were found in each isolate that were previously identified by transposon mutagenesis (Table 3). Subsequently, the identified mutant alleles were introduced into the ancestral PAO1 strain, producing the RSCV phenotype (S5 Fig). All these genotypes overproduced PelC (S6A Fig) and Psl (S6B Fig). The most frequent RSCV-linked mutations occurred in genes encoding flagellum biosynthesis and function. Mutants bearing these alleles had a frequency from 23% to 63% in each of the replicate biofilm populations (Table 3). Altogether, these observations indicate that EPS-overproducing flagellar mutants reproducibly evolve during *P*. *aeruginosa* biofilm growth.

**Table 3:** Identity and frequency of RSCV-linked alleles in model biofilms.

Reactor	Population size (10^10^ CFU/reactor)	Est. RSCV frequency	RSCV-linked mutation(s)^1^	Description	Est. allele frequency (*a*/*n*)^2^
1	10.54 ± 0.05	0.51	*fliG*_128T>G_ (V43G)	flagellum motor switch protein	0.22 (15/34)
			*fliG*_834_844ΔGAAGGTCTTCA_	flagellum motor switch protein	0.12 (8/34)
2	10.79 ± 0.03	0.55	*fliM*_718C>T_ (Q240*)	flagellum motor switch protein	0.23 (40/94)
3	10.53 ± 0.03	0.46	*wspF*_777C>A_ (S259R)	probable methylesterase	0.01 (1/72)
4	10.64 ± 0.08	0.60	*tpbB*_668A>G_ (D223G)	diguanylate cyclase	0.01 (2/98)
			*fliH*_178G>T_ (E60*)	putative flagellum TTSS protein	0.33 (54/98)
5	10.47 ± 0.05	0.73	*fliM*_915delC_	flagellum motor switch protein	0.63 (49/57)

^1^Nomenclature extensions to describe complex mutations adapted from Dunnen and Antonarkakis [83].

^2^*n* denotes the number of RSCVs isolated in a random sample from the reactor, and *a* denotes the number of those RSCVs that had the indicated RSCV-linked allele, which was determined by targeted sequencing of the allele in each isolate. Allele frequency in the biofilm population, therefore, was estimated as the ratio of *a*/*n*.

### EPS overproduction provides flagellar mutants with a biofilm fitness advantage

**Fig 5:**
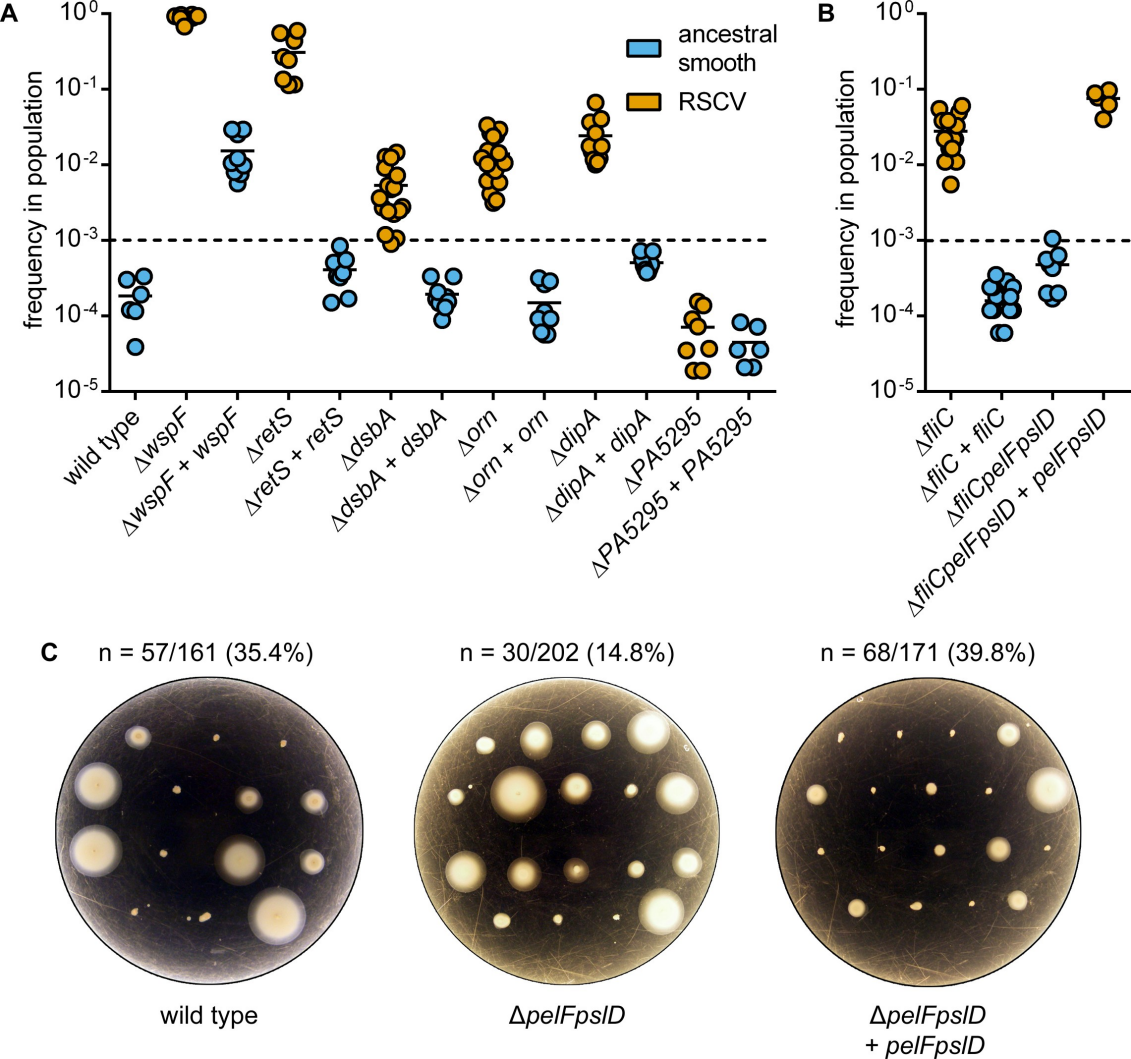
The biofilm fitness advantage of flagellar mutants depends on Pel and Psl. (A) Frequency of RSCV cell lines after 3 days of co-culture in biofilm reactors with the ancestral PAO1 strain. Datum points represent technical replicates from each of three independent drip-flow reactors. (B) Frequency of the Δ*fliC*, Δ*fliC*Δ*pelF*Δ*pslD* and complemented strains after 3 days of co-culture in biofilm reactors with the ancestral PAO1 strain. Datum points represent technical replicates from each of three independent drip-flow reactors. (C) Frequency at which motility was lost during laboratory evolution of wild type, Δ*pelF*Δ*pslD* and complemented strains when grown for 5 days in a drip-flow reactor. The hatched line indicates the starting frequency of the mutant cells in the drip-flow reactors. Orange points represent strains that can express the RSCV phenotype, blue points represent strains with ancestral smooth colony morphology.

Competitive co-culture was used to assess the fitness of precisely defined genotypes in laboratory-grown biofilms. Here we used a starting ratio of 1 mutant or complemented cell to 1000 ancestral cells. Viable cell counting was enabled by genetically tagging bacteria with an antibiotic resistance gene (*aacC1*). The *aacC1* gene is not selectively neutral, as the wild type PAO1 strain labelled in this manner decreased in frequency approximately 6-fold during competition with its antibiotic-sensitive ancestor (Fig 5A). To account for this change in fitness, all changes in frequency of *aacC1*-labelled mutant cell lines were calculated relative to the frequency of the *aacC1*-labelled wild type strain. Correlating with previous reports and with only a single exception (Δ*PA5295*), we found that acquisition of an RSCV-linked mutation was strongly associated with an increase in biofilm fitness (Fig 5A and 5B).

Subsequently, we focused on flagellar mutants in order to disentangle the roles of EPS production and loss of flagellar function to biofilm fitness. During the course of co-culture, the Δ*fliC* mutant increased in frequency 150-fold and genetic complementation eliminated the increased biofilm fitness of this genotype (Fig 5B). In contrast to Δ*fliC*, a Δ*fliCpelFpslD* triple mutant had greatly diminished biofilm fitness, increasing in frequency only 2.5-fold relative to the *aacC1*-labelled control (Fig 5B). Genetic complementation of this triple mutant with single copies of *pelF* and *pslD* expressed from their native promoters restored the biofilm fitness of this strain, which increased ~410-fold in frequency during co-culture (Fig 5B). These data suggest that Pel and/or Psl are essential for the biofilm fitness of a flagellar mutant. To further test this interpretation, we directly tested for the loss of swimming motility during experimental evolution of wild type and Δ*pelFpslD* strains in drip-flow reactors (Fig 5C). After 5 days, 35.4% of the biofilm isolates from wild type and 14.9% from the Δ*pelFpslD* strain were negative for swimming motility. By contrast, repeating experimental evolution with the Δ*pelFpslD* mutant that had been complemented with *pelF* and *pslD* yielded a population where 39.8% of biofilm isolates were negative for swimming motility. Overall, these results suggest that EPS expression is a gain-of-function phenotype that significantly contributes to the biofilm fitness of flagellar mutants.

### Flagellar mutants and other classes of RSCVs described here are found among CF isolates

**Fig 6:**
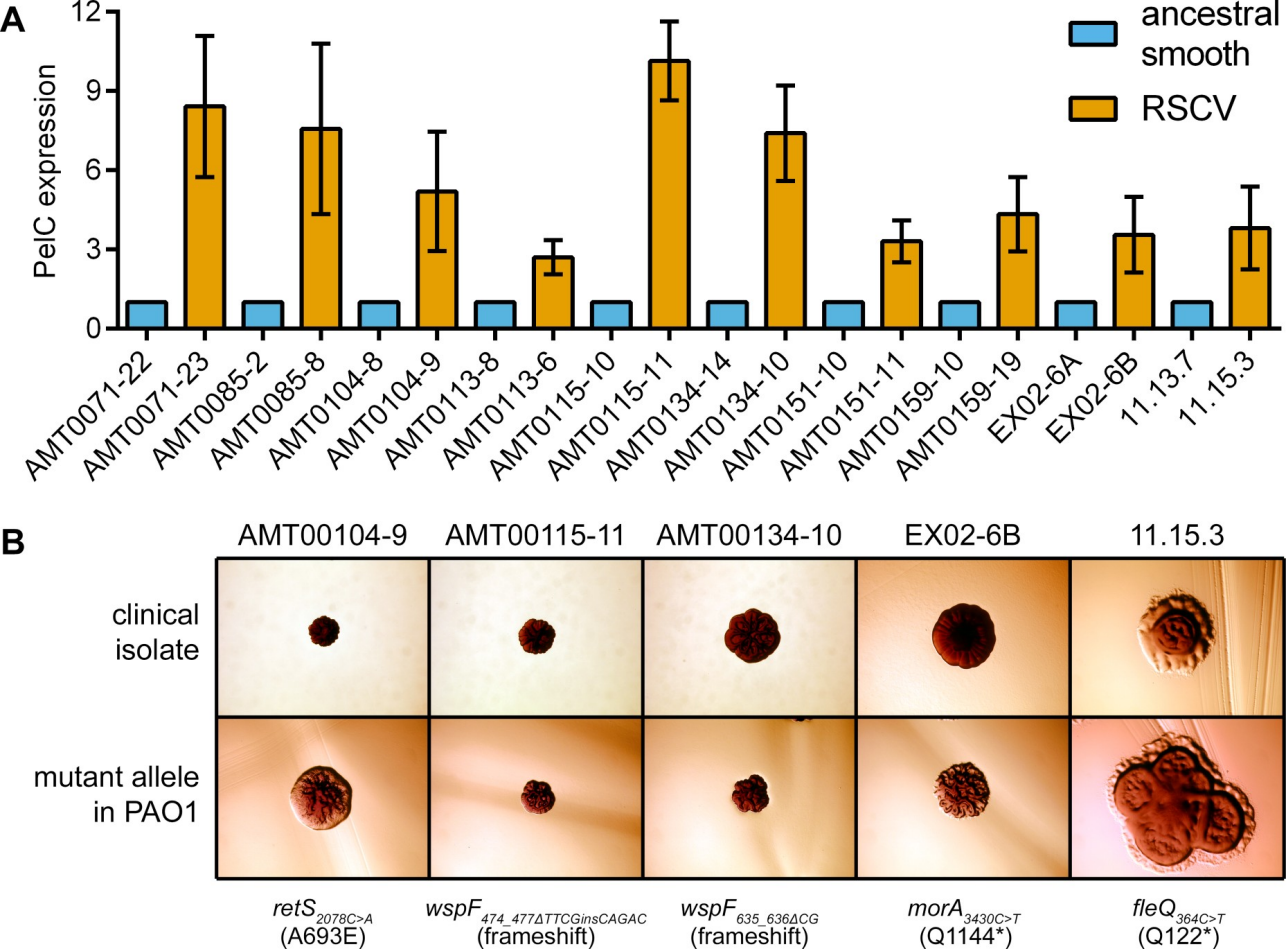
Multiple genes are linked to the RSCV phenotype of clinical isolates and are associated with PelC overexpression. (A) Semi-quantitative Western blots for PelC from strains grown on VBMM agar. Each bar indicates the mean and SD for 3 independent biological replicates. (B) Colony morphology of clinical isolates recovered from CF patients. RSCV-linked alleles that were identified in the clinical isolates by genome sequencing were introduced into the *P*. *aeruginosa* PAO1 strain. In all panels, bacteria were cultured and photographed on VBMM-based agar containing Congo red and brilliant blue R (see Material and Methods). Each panel represents an area that is approximately 5.0 mm × 3.5 mm.

*P*. *aeruginosa* that have lost flagellar motility have been reported among CF isolates [8, 68]. Here we examined clonally related isolates from previous studies of *P*. *aeruginosa* genetic diversity in CF respiratory infections [2, 40, 69]. Initially, we identified eight closely related pairs of isolates (Fig 6A and S1 Table, each pair recovered from a different, individual CF patient) in which one isolate had smooth colony morphology and the other displayed an RSCV phenotype on VBMM (not on LB; S7 Fig). An additional two pairs of isolates were identified among early and late isolates from prior [2] genome sequencing projects. All the RSCVs displayed upregulation of PelC relative to their related strains with smooth colony morphology (Fig 6A). Data from directed [2] as well as *de novo* genome sequencing was used to identify putative RSCV-linked mutations. In five cases we successfully identified mutations (*retS*_*2078C>A*_, *wspF*_*474_477ΔTTCGinsCAGAC*_, *wspF*_*635_636ΔCG*_, *morA*_*3430C>T*_, and *fleQ*_*364C>T*_), that when introduced into PAO1, caused the RSCV phenotype (Fig 6B). We were unsure as to why the *wspF* mutant allele was smooth on LB for two of these clinical isolates. We postulate that this difference could be due to an epistatic interaction in the genetic background of the CF isolates. Regardless, these findings indicate that CF RSCV isolates are associated with similar gain-of-function phenotypes (EPS overexpression), notably including a flagellar mutant.

### A new way to interpret the fitness benefits of flagellar mutations

**Fig 7:**
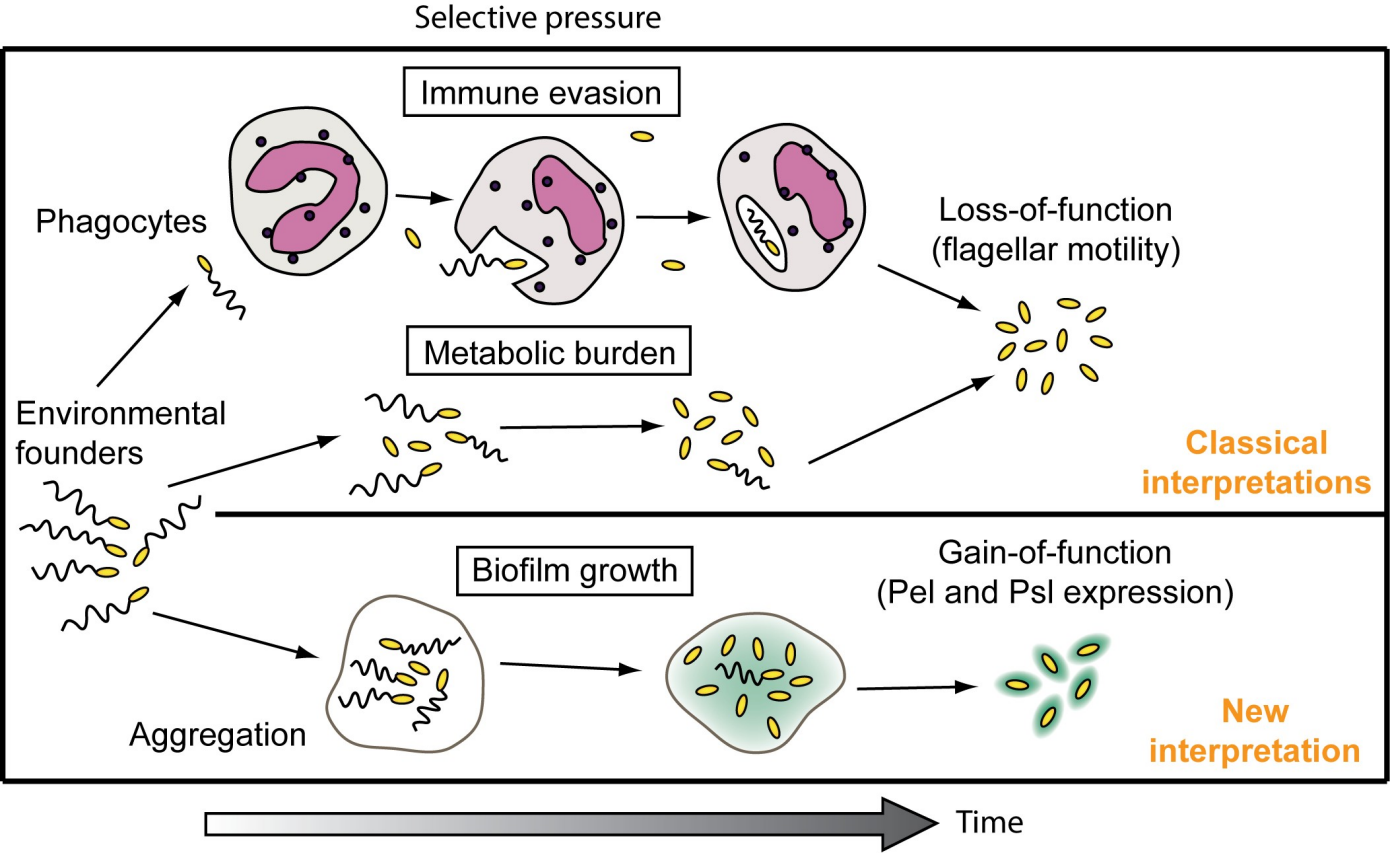
Interpreting the benefits of loss of flagellar function in laboratory biofilms and CF. The classic interpretations for loss of the flagellum view evasion of the host immune system and a decreased doubling time as fitness benefits that could lead to the evolution of non-flagellated bacteria during infection. We propose that selection for gain-of-function phenotypes related to exopolysaccharide production offers a new way for interpreting the causes and consequences of flagellar gene mutation in biofilms and CF infection.

Because an accurate and established small animal model for CF respiratory infection is not available, it is difficult to evaluate what selective forces are at work *in vivo*. Several factors could lead to bacterial diversification in CF airways, and these pressures might include immune functions, fluctuations in antibiotics, O_2_ tension or nutrient availability, among others. *P*. *aeruginosa* non-mucoid isolates that have lost their flagella have been reported for at least thirty-five [8, 68] years and there are two classic interpretations for the benefits of this adaptation. First, loss of the flagellum confers immune evasion, which could allow *P*. *aeruginosa* flagellar mutants to escape negative selection by host immune cells [70]. Second, mutation of flagellar genes could afford a metabolic benefit to mutant cells by eliminating the energetic cost of synthesizing the flagellum, which could provide flagellar mutants with an increased growth rate. Our discovery that flagellar mutants have a gain-of-function phenotype–Pel and Psl expression–offers another perspective on the selective pressures that amplify flagellar gene mutations (Fig 7). One possibility is that positive selection for aggregation and biofilm growth could favor loss of the flagellum; alternatively, immune selection or an increased growth rate could lead to the evolution of non-flagellated bacteria that are also particularly successful at forming biofilms. Because there are multiple parameters that may drive expression of the RSCV phenotype on VBMM agar but not on LB, which might include, for example, nutrition-dependent modulation of c-di-GMP signaling enzymes [71, 72], we have yet to decipher the molecular mechanism for this phenomenon. However, we observed that flagellar mutants display an RSCV phenotype and overexpress PelC and Psl on synthetic cystic fibrosis sputum medium (SCFM) agar (S8 Fig). Staudinger and colleagues have also observed that flagellar mutants aggregate in mucus-based gels and that aggregation decreases the susceptibility of *P*. *aeruginosa* flagellar mutants to antibiotics [73]. We remark that our interpretation for how these gain-of-function phenotypes might provide benefits during infection may not be exclusive of other classic interpretations for the benefits of the loss of flagella. Nevertheless, because Pel and Psl production are associated with biofilm fitness, we propose that loss of the flagellum may lead to biofilm growth that contributes to increased bacterial persistence during infection.

## Materials and methods

### Bacterial strains and isolates, plasmids and growth conditions

Bacterial strains or isolates and plasmids are listed in S1 Table and S2 Table, respectively. Collection of all clinical isolates was approved by the Seattle Children’s and/or University of Washington Hospital Institutional Review Boards (IRBs). *P*. *aeruginosa* was grown at 37°C in lysogeny broth (LB, 10 g L^-1^ tryptone, 5.0 g L^-1^ yeast extract, 5.0 g L^-1^ NaCl) or Vogel-Bonner Minimal Media (VBMM; 0.2 g L^-1^ MgSO_4_•7H_2_O, 2.0 g L^-1^ citric acid, 3.5 g L^-1^ NaNH_4_HPO_4_•4H_2_O, 10 g L^-1^ K_2_HPO_4_, pH 7.0) [74] with 10 mM citrate. VBMM with Congo red (CR), brilliant blue R (BB) and 1.0 g L^-1^ casamino acids (VCBA) was used to grow clinical isolates. Semi-solid plate media were prepared by adding 1.5% w/v Bacto agar to LB or 1.0% noble agar to VBMM. Additional details of growth conditions, antibiotic selection and plasmid construction are described in S1 Text.

### Digital photography

An Olympus SZX-ILLK100 stereomicroscope equipped with a C-7070 wide zoom digital camera was used to photograph colonies growing on agar media. Pictures of Petri plates were captured using a Pentax Optio W10 digital camera with tripod. Digital images were adjusted for contrast and brightness using Photoshop CS6 (Adobe).

### Transposon mutagenesis

Established protocols were used for mutagenesis of *P*. *aeruginosa* with the transposon miniTn5-Pro [42]. Transconjugants were selected on VBMM plates containing 100 μg/ml gentamicin (Gm) + CR/BB. This selection was repeated on plates that additionally contained 0.2% arabinose. Transposon insertion sites were identified by plasmid rescue of the flanking genomic regions followed by DNA sequencing (see S1 Text).

### Construction of deletion and site-directed mutants

Deletion alleles were assembled *in vitro* by removing an in-frame fragment of coding sequence from each gene. This was done by joining PCR products amplified from the adjacent regions of the chromosome by splicing by overlapping extension (SOE) PCR. These DNA fragments were then cloned by restriction into the suicide vector pEX18Gm. Alternatively, cloning of deletion alleles was carried out using Gateway recombination with the suicide vectors pDONRPEX18Gm or pEX18GmGW as previously described [75] (see S1 Text). Alleles containing point mutations that evolved in either biofilm or clinical isolates were directly cloned by PCR using primers specific for the target open reading frames (ORFs) and then introduced into allelic exchange vectors using Gateway technology (see S1 Text). Unmarked deletion and site-directed mutations were then introduced into the *P*. *aeruginosa* chromosome by two-step allelic exchange using established procedures [75].

### Complementation analysis

PCR primers (S3 Table) targeting wild type alleles and their native promoters were tailed with *attB* sequences and these PCR products were then cloned by Gateway BP recombination into pDONR223. The plasmid inserts were sequenced using universal forward and reverse M13 primers (S3 Table) and then transferred by LR recombination into pUC18-miniTn7T-Gm-GW [76]. For the purpose of complementation analysis with two ORFs, we used the plasmid pUC18-miniTn7T2.1-Gm-GW [77] and two divergently transcribed ORFs were assembled in multiple steps using SOE-PCR and Multisite Gateway cloning technology (see S1 Text). The miniTn7 constructs were then introduced into the *P*. *aeruginosa* chromosome via electroporation with the helper plasmid pTNS1 [78]. Insertion at the neutral *attTn7* adjacent to *P*. *aeruginosa glmS* was confirmed by PCR using the primers PTn7L, PTn7R, Gm-up and Gm-down (S3 Table) as previously described [76].

### Drip-flow biofilm reactors

*P*. *aeruginosa* biofilms were grown in drip-flow reactors using 1% tryptic soy broth according to standard protocols [79]. Biofilms were recovered using a cell scraper to dislodge biomass into sterile PBS and these were then disrupted using a tissue homogenizer. Biofilm cells were serially diluted ten-fold in PBS and then plated onto either LB or VBMM + CR/BB agar.

### Genome sequencing and bioinformatics

Illumina sequencing technology was used to collect deep-coverage genome sequence data. The procedures for library construction, sequencing, genome assembly and SNP analysis are detailed in S1 Text. Sequencing data have been deposited with links to BioProject accession number PRJNA625996 in the National Center for Biotechnology (NCBI) BioProject database (https://www.ncbi.nlm.nih.gov/bioproject/).

### Immunoblots

Chemiluminescent Western and dot blots for PelC and Psl, respectively, were carried out by established methods [80] with slight modification (see S1 Text). Horse-radish peroxidase linked secondary antibodies were used in conjunction with the Pierce SuperSignal West Pico ECL reagent (Thermo Scientific) to visualize proteins bound by primary antibodies and Chemiluminescence was captured and quantified using a FluorChem Q (Alpha Innotech).

### ELISA assays

ELISA assays were carried out according to the method of Byrd and colleagues [80] with slight modification using a human monoclonal anti-Psl antibody, Cam-003 (MedImmune) [81] (see S1 Text).

### Motility assays

The swimming medium was VBMM that contained 0.3% w/v bacto-agar (Difco). Swim plates were stab inoculated with bacteria from an LB or VBMM agar culture grown overnight at 37°C using a sterile toothpick. Plates were then incubated at 37°C for 16–18 h and photographed.

## Supporting information

S1 Text Supplementary materials and methods and supplementary references. (PDF)

S1 Table Bacterial strains. (PDF)

S2 Table Plasmids. (PDF)

S3 Table Primers and sequencing adapters. (PDF)

S1 Fig *P*. *aeruginosa* strains with nutrition-dependent RSCV-linked genotypes overexpress PelC and/or Psl even when grown under conditions where the RSCV phenotype is not expressed. (A) Semi-quantitative dot blots for the Psl polysaccharide from strains grown on VBMM agar. (B) Semi-quantitative Western blots for PelC from strains grown on VBMM agar. (C) Semi-quantitative dot blots for the Psl polysaccharide from strains grown on LB agar. (D) Semi-quantitative Western blots for PelC from strains grown on LB agar. Each bar indicates the mean and SD for 1 or 2 technical replicates from each of 3 to 4 independent biological replicates.
(TIF)

S2 Fig Nutrition-dependent RSCVs do not require WspR and have smooth, and in some instances small, colony morphology on LB agar. (A) The diguanylate cyclase WspR is dispensable for the nutrition-dependent RSCV phenotype resulting from RSCV-linked mutations identified in this study. Bacteria in these panels were cultured and photographed on VBMM agar containing Congo red and brilliant blue R (see Material and Methods). (B) These nutrition-dependent RSCV-linked genotypes give rise to bacterial colonies with smooth morphology on LB agar. LB agar was also supplemented with the dyes Congo red and brilliant blue R. Each panel represents an area that is approximately 5.0 × 3.5 mm.
(TIF)

S3 Fig Only some deletion mutations in flagellar operons are linked to the *P*. *aeruginosa* RSCV phenotype. Precisely defined in-frame deletion mutations were introduced into a series of flagellar genes in wild type PAO1 strain. Only some of these mutations caused the RSCV phenotype. In all panels, bacteria were cultured and photographed on VBMM agar containing Congo red and brilliant blue R (see Material and Methods). Each panel represents an area that is approximately 5.0 mm × 3.5 mm.
(TIF)

S4 Fig Overexpression of PelC by some flagellar mutants is surface-contact dependent. Semi-quantitative Western blots for PelC from flagellar mutants grown (A and B) in shaken LB or VBMM cultures, or (C and D) on the surface of LB or VBMM agar, respectively. Each bar indicates the mean and standard deviation for 1 or 2 technical replicates from each of 3 to 4 independent biological replicates.
(TIF)

S5 Fig Mutant alleles that evolved during biofilm growth are linked to the RSCV phenotype. Photographs of agar-grown RSCVs that were isolated from biofilm reactors after experimental evolution. RSCV-linked mutations were identified by whole genome sequencing (top). Colony morphology of mutants in which the RSCV-linked allele from the biofilm isolate was introduced into the ancestral PAO1 strain (bottom). In all panels, bacteria were cultured and photographed on VBMM agar containing Congo red and brilliant blue R (see Material and Methods). Each panel represents an area that is approximately 5.0 mm × 3.5 mm.
(TIF)

S6 Fig Biofilm-evolved flagellar mutant genotypes overexpress PelC and Psl. (A) Semi-quantitative PelC Western blots for strains grown on VBMM agar. (B) Semi-quantitative Psl dot blots for strains grown on VBMM agar. Each bar indicates the mean and standard deviation for 3 to 6 independent biological replicates.
(TIF)

S7 Fig Nutrition-dependent colony morphology of closely related pairs of CF isolates. (A) Colony morphology of isolates on LB and VBMM agar. Each panel represents an area that is approximately 5.0 mm × 3.5 mm. (B) Analysis of pulsed-field gel electrophoresis (PFGE) data indicating that pairs of CF isolates with smooth and RSCV colony phenotypes are close genetic relatives. Phylogenetic relationships were calculated from existing PFGE data for the PASA collection of *P*. *aeruginosa* isolates at Seattle Children’s Hospital [40] using Bionumerics Seven (Applied Maths).
(TIF)

S8 Fig Flagellum mutants overexpress extracellular polysaccharides and display the RSCV phenotype on synthetic cystic fibrosis sputum medium (SCFM) agar. (A) Semi-quantitative Western blots for PelC from strains grown on SCFM agar. (B) Semi-quantitative dot blots for the Psl polysaccharide from strains grown on SCFM agar. (C) Colony morphology of flagellum mutants on SCFM agar. Each panel represents an area that is approximately 5.0 mm × 3.5 mm. Each bar indicates the mean and SD for 3 biological replicates. Relative expression levels have been normalized to the Δ*wspF* strain.
(TIF)
