# Supplementary material for: Elevated exopolysaccharide levels in Pseudomonas aeruginosa flagellar mutants have implications for biofilm growth and chronic infections
Source: PLoS Genet. 2020 Jun 12;16(6):e1008848. doi: 10.1371/journal.pgen.1008848 (PMC7314104; doi:10.1371/journal.pgen.1008848)
Supplement: S1 Text — (PDF) [file pgen.1008848.s001.pdf]

S1 Text

Supplementary Materials and Methods

Additional bacterial strains and growth conditions

All *E. coli* strains (S1 Table) were propagated on LB medium. Where appropriate, antibiotics were added to maintain or select for plasmids as follows: for *E. coli*, ampicillin (Ap) at 100 µg/ml, gentamicin (Gm) at 10 µg/ml, kanamycin (Kn) and spectinomycin (Sp) at 50 µg/ml; for *P. aeruginosa*, carbenicillin (Cb) at 300 µg/ml and Gm at 100 µg/ml. Where indicated, the dyes congo red (CR) and brilliant blue R (BB) were added to media at 40 µg/ml and 15 µg/ml, respectively.

Synthetic cystic fibrosis sputum medium (SCFM) agar was prepared using the SCFM recipe described by Palmer and colleagues [1] with a few key modifications. Briefly, to make the buffered base solution, 0.122 g NH_4Cl, 1.114 g KCl, 3.03 g NaCl, and 2.0926 g of MOPS were added to 180 mL of milliQ purified water. To this solution, 6.5 mL of 0.2 M NaH_2PO_4, 6.25 mL of 0.2 M Na_2HPO_4, 0.348 mL of 1 M KNO_3, and 1.084 mL of 0.25 M K_2SO_4 were added. In a separate flask, 15 g noble agar were added to 600 mL of milliQ water. The agar solution was autoclaved and then cooled to 65 °C in a water bath. Concomitantly, the 19 amino acids stocks used by Palmer and colleagues [1], which were sterilized using 0.22 µm polyethylene sulfone (PES) syringe filters, were added to the buffered base solution at the concentrations previously reported [1]. This solution was adjusted to pH 6.8 and then filter sterilized with a 0.22 µm bottle top filter. Next, the buffered solution containing amino acids was warmed to 65 °C and then combined with the molten noble agar. Finally, the following filter sterilized components were added to the medium: 1.754 mL I M $CaCl_2$, 0.606 mL 1 M $MgCl_2$, 1 mL 3.6 mM $FeSO_4$, 3 mL 1 M D-glucose, and 9.3 mL 1 M L-lactate. The SCFM agar was then dispensed into standard Petri dishes.

Transposon mutagenesis

The transposon miniTn5-Pro was delivered on the suicide vector pUT from *E. coli* S17.1 into *P. aeruginosa* by standard mating techniques [2]. After mating, donor and recipient cells were suspended in 1.0 ml of phosphate buffered saline (PBS, pH7.2). Transconjugants were selected by plating 500 µl of this suspension on VBMM agar plates containing 100 µg/ml Gm + CR/BB. The remaining 500 µl was spread on plates that additionally contained 0.2% arabinose. Colony morphology was examined using a stereomicroscope. Colonies with the desired morphology were picked, streaked on selective medium and then photographed.

To determine the site of miniTn5-Pro transposition, genomic DNA (gDNA) was purified, digested with NcoI, and then ligated under dilute conditions to favor intramolecular ligation. The ligation mixture was desalted, transformed into *E. coli* EC100 (λpir) and then plated onto LB agar plates containing 10 µg/ml Gm. Plasmids were isolated from the colonies that arose, and the DNA was sequenced using the para2 primer (Table S3) as previously described [3]. The chromosomal location was then determined by BLAST [4] analysis of the sequence against the *P. aeruginosa* PAO1 genome using the *Pseudomonas* genome database [5].

Construction of allelic exchange vectors using Gateway technology

All plasmids and primers used in genetic manipulations are listed in S2 Table and S3 Table, respectively. Deletion alleles were assembled by removing an in-frame segment of coding sequence, constituting >95% of the gene, from the target open reading frame (ORF) as previously detailed [6]. Briefly, two PCR products, which were amplified using primers that targeted the adjacent upstream and downstream regions of the chromosome flanking the target ORF, were joined through splicing by overlapping extension (SOE) PCR. The upstream forward and downstream reverse primers used to generate these deletion alleles were tailed with *attB1* or *attB2* sequences as described in the Gateway Cloning Technology Manual (Invitrogen) and, using

Gateway technology, the deletion allele was then recombined with either pDONR221 or pDONR223 using BP Clonase II (Invitrogen). Alternatively, ORFs containing a point mutation were amplified using forward and reverse primers tailed with *attB1* and *attB2* sequences, respectively, then recombined with either pDONR221 or pDONR223. All these entry plasmids were then sequenced using M13F and M13R primers. Finally, the mutant alleles from these entry vectors were recombined with pEX18GmGW using LR Clonase II (Invitrogen) to create the desired allelic exchange vectors.

To enable one-step construction of allelic exchange vectors using Gateway technology, we built the plasmid pDONRPEX18Gm. A 2.5 kb DNA fragment, containing the Gateway donor site from pMK2010, was cloned using primers JJH469 and JJH470 (S3 Table). The PCR product was then restricted with HindIII and ligated into the HindIII site of pEX18Gm. The resulting plasmid, pDONRPEX18Gm, thus has the 5'-*att*P2-*ccdB*-*cat*-*att*P1-3' cassette inserted into the multiple cloning site and in between the M13F and M13R universal priming sites. PCR products (containing mutated alleles) that had been tailed with *attB1* and *attB2* sequences were then recombined with pDONRPEX18Gm using BP Clonase II, and then sequenced using M13F and M13R primers. The products of these recombination reactions were thus entry vectors that could be used directly for allelic exchange.

Construction of miniTn7 vectors using multisite Gateway technology

Complementation constructs containing two divergently transcribed ORFs were assembled in multiple steps using SOE-PCR and Multisite Gateway Cloning technology (Invitrogen). To create a complementation construct for *pslD* and *pelF*, the native promoters of the *pslD* and *pelF* genes, along with the start codon and first 15 nucleotides of the first gene in each operon, were cloned by PCR. At the same time, the *pslD* and *pelF* ORFs were cloned using PCR primers that also targeted the last 15 nucleotides of the upstream ORFs (S3 Table), which preserved the native

ribosomal binding sites of these two genes. The promoter and ORF fragments were then fused using SOE-PCR with *attB* tailed primers. This generated *attB*5r-*pslA*$_{Pro}$::*pslD*-*attB*1 and *attB*5-*pelA*$_{Pro}$::*pelF*-*attB*2 fragments. These SOE-PCR products were then recombined into the vectors pDONR221 P1-P5r and pDONR221 P5-P2, respectively, using BP Clonase II. These entry vectors were sequenced using M13F and M13R primers, and then assembled into the destination vector pUC18-miniTn7T2-Gm-GW using LR Clonase II Plus (Invitrogen) to create pJJH149.

Western blots

Whole cell lysates were prepared from *P. aeruginosa* strains grown on LB or VBMM + CR/BB agar for 24 h at 37 °C. Cells were collected using a polyester-tipped swab, suspended in PBS, then collected by centrifugation. Alternatively, cells were collected by centrifugation from 1.0 ml of an overnight culture. Cell pellets were then solubilized in Laemilli buffer by heating at 100 °C for 15 min. Protein concentration was measured using the Pierce 660 nm protein assay with ionic detergent compatibility reagent (Thermo Scientific). 250 or 1000 ng of total protein was loaded in each lane of a precast 12.5% TRIS-glycine gel for SDS-PAGE (Biorad). Following electrophoresis, protein was transferred to a nitrocellulose membrane. Non-specific binding sites were blocked by soaking the membrane in 5.0% non-fat milk in TRIS-buffered saline containing 0.1% Tween-20 (TBST). Membranes were probed with a polyclonal, rabbit α-PelC antibody (diluted 1:5000) that had been adsorbed against *P. aeruginosa* whole cell lysate by standard protocols. These blots were washed three times with TBST, and then probed with a secondary α-rabbit horseradish peroxidase-conjugated antibody (diluted 1:25000, Thermo Scientific). Following three additional washes with TBST, membranes were incubated with the Pierce SuperSignal West Pico ECL reagent according to the manufacturer's directions (Thermo Scientific). Chemiluminescence was imaged and quantified using a FluorChem Q (Alpha Innotech).

Whole cell lysates for clinical isolates were prepared identically to those described above for *P. aeruginosa* PAO1 strains, except bacteria were grown on VBMM agar + CR/BB for 48 h at 37°C. Following transfer of proteins from clinical specimens to nitrocellulose, the membrane was cut in half and probed with either anti-PelC antibodies as described above, or with mouse anti-RNA polymerase (RNAP) antibodies (Neoclone Biotechnologies) according to manufacturer's directions. Imaging and quantification was carried out as described above.

Anti-Psl dot blots

Psl immunoblots were performed as previously described[7] but with several modifications. Cells were gathered from the surface of LB or VBMM + CR/BB agar plates using a sterile polyester-tipped swab and were then suspended in 0.5 M EDTA. Alternatively, cells were collected by centrifugation from 1.0 ml of an overnight culture and the pellets were then suspended in 250 µl of 0.5 M EDTA. These cell suspensions were heated at 100 °C for 60 min. Cell debris was removed by centrifugation and the protein concentration of the supernatant was determined using the Biorad protein assay. Samples were next treated with 10 µl proteinase K (QIAGEN, 600 mAU ml^{-1}) per 250 µl of 0.5 M EDTA at 56°C for 30 min. Proteinase K was then heat inactivated for 15 min at 100°C. For each strain, a total of 50, 100 or 250 ng of protein was loaded per 5 µl test spot on a nitrocellulose membrane. Chemiluminescence was imaged and quantified using a FluorChem Q (Alpha Innotech) and ImageJ analysis.

Anti-Psl enzyme-linked immunosorbent assay (ELISA)

Bacteria were collected from the surface of a VBMM agar plate culture using a cotton swab and then diluted to an OD_{600} = 0.05 in sodium carbonate buffer. A total of 100 µl of this cell suspension and 100 µl of sodium carbonate buffer were added to each well of a microtiter plate, which was sealed and incubated at 37°C for 2 h. The wells of the plate were then washed three times with 200 µl PBS containing 0.05% Tween (PBST). Next, 200 µl of 5% non-fat dry milk (NFDM) (Difco)

and PBST were added to each well, and incubated at 37°C for 30 min. Primary antibody was added at a 1:5,000 dilution in 5% NFDM/PBST, and the plate was incubated at 37°C for 30 min. The wells of the plate were then washed five times with 200 µl PBST. Secondary antibody (horseradish peroxidase linked human antibody) was applied at a 1:3,000 dilution, and microtiter plates were incubated at 37°C for 30 min. The wells of the plate were then washed five times with 200 µl PBST. Finally, 100 µl of SuperAqua Blue ELISA Substrate (eBioscience) were added to each well of the microtiter plate, and incubated at 25°C for 30 min. Substrate oxidation was quantified by measuring OD_{405} with a microtiter plate reader.

Biofilm cultivation and competition assays in drip-flow reactors

Drip-flow reactors were assembled with stainless steel coupons and sterilized according to the manufacturer's directions (Biosurface Technologies). Starting with cryogenic stocks, the desired bacterial strain was streaked out twice on VBMM agar (with antibiotic selection, where appropriate), and an inoculum was prepared by suspending colonies from the second agar subculture in phosphate buffered saline (PBS, pH 7.4) to match a 1.0 Mcfarland Standard (an OD_{600} of 0.26, or approximately 3.0×10^{8} CFU/ml). A 1.0 ml aliquot from this standard was then diluted into 9.0 ml of 10% tryptic soy broth (TSB) and subsequently, the number of starting cells was verified by viable cell counting (4 × 20 µl aliquots). The remainder of this standardized inoculum was then added to the sealed aluminum chamber of a drip-flow reactor and, to allow attachment, incubated overnight on a level surface at room temperature (25 °C). The next morning the reactor was tilted on a block at a 10° incline and drained. The reactors were then moved to a warm-room (at 37 °C) and connected to a pre-warmed medium reservoir with silicone tubing as previously described [8]. Subsequently, the silicone tubing was inserted into the cartridge of a Watson-Marlow 8-channel peristaltic pump, and drip-flow was initiated by setting the pump to a velocity of 3.75 rotations per minute. After 5 d, biofilms were harvested into 8 ml of PBS, homogenized, serially diluted, and then scored for viable cell counts and colony morphology.

For biofilm competition assays, a standardized inoculum was prepared for each strain as described above, except 999 µl of the ancestor and 1 µl of a competing mutant or complemented strain were added to 9.0 ml of 10% TSB. Viable cell counts were carried out and drip-flow reactors were inoculated the same as before, except drip-flow was maintained for 3 d at 37 °C. Biofilms were harvested into 8 ml of PBS, homogenized, serially diluted, and then scored for viable cell counts and colony morphology on agar with and without gentamicin selection. This facilitated direct viable cell counting of the *aacC1*-tagged biofilm competitors.

Library preparation for genome sequencing of evolved PAO1 strains

For each genome a random-fragment library was constructed using a custom paired-end protocol [9]. Genomic DNA (gDNA) was sheared using a Bioruptor UCD-200 (Diagenode) and end repaired using an End-It DNA end repair kit (Epicentre). Repaired fragments were subjected to A tailing using Taq DNA polymerase (Roche). Custom Y adaptors were used to barcode strain specific gDNA (S3 Table), and these were added by using T4 DNA ligase (New England Biolabs). These custom adaptors contained defined sequences that were read as the first 13 bases. In addition to 9 unique bases, each barcode contained 4 identical bases at the 3' end to reduce variation in ligation efficiency (S3 Table). Libraries were size selected by using automated electrophoresis on a Pippen Prep system (Sage Science) and assessed for size range and concentration using a Qubit fluorometer (Invitrogen) and a Bioanalyzer (Agilent). Sequencing was done on GAIIx genome analyzer (Illumina) with Data Collection Software version SCS 2.6 according to the manufacturer's protocols. Eight samples were pooled at equimolar concentration and sequenced in one lane of an Illumina version 4 single read flow cell. A duplicate lane of sequencing was added to ensure sufficient coverage.

Read processing and variant discovery for evolved PAO1 strains

The Burrows-Wheeler Alignment (bwa v0.6.1) [10] software tool was used to align reads from sequencing to the PAO1 reference genome [5]. The SAMtools mpileup method (SourceForge) was used to generate a list of single-nucleotide polymorphisms (SNPs). We used a custom script to identify each SNP as nonsynonymous or synonymous and as genic or intergenic [11]. We narrowed the SNP list to changes that were present in RSCVs but not in the resequenced PAO1 ancestor. Remaining SNPs were verified by using the Integrated Genomic Viewer (IgV v1.5) [12].

Library preparation for de novo genome sequencing of clinical isolates

Sequencing libraries were prepared by transposase-catalyzed fragmentation and adaptor ligation using the Nextera DNA Sample Preparation Kit (Epicentre). Transposition reactions contained 50 ng gDNA in a volume of 5 μl, with 0.1 μl transpose enzyme and 1 μl 5 × Buffer HMW. Real-time PCR was used to amplify each library with a unique 9-mer barcoded adaptor[13], using the FailSafe PCR kit with Buffer K (Epicentre) and SYBR Green I at 0.25 × final concentration. Equal volumes of each library were pooled for sequencing across three partial lanes (in combination with non-bacterial libraries) on an Illumina HiSeq 2000 using paired 101 bp reads with a 9 bp index read for demultiplexing libraries.

Read processing and variant discovery for clinical isolates

Reads were demultiplexed allowing up to 2 edits to barcode sequences, and aligned to the PAO1 reference assembly using bwa v0.6.1 [10]. Reads aligned to overlapping positions were merged and flanking adaptor sequences were trimmed using Smith-Waterman alignment implemented in python. Single-base and indel variant calls were made with GATK v1.6.5 [14], and each library was then genotyped against the full set of variants found across all libraries. Candidate mutations between each pair of strains were defined as sites covered by at least 8 reads in each strain, with the variant allele present at greater than or equal to 50% frequency among reads from one strain (relative to PAO1) but less than 25% frequency in the paired strain. Copy number variants

between paired strains were identified by taking the log-ratio of read depths from each pair of strains across 100 bp windows tiling the PAO1 reference.

Supplementary References

1. Palmer KL, Aye LM, Whiteley M. Nutritional cues control *Pseudomonas aeruginosa* behaviour in cystic fibrosis sputum. J Bacteriol. 2007;189(22):8079-87.

2. de Lorenzo V, Timmis KN. Analysis and construction of stable phenotypes in Gram-negative bacteria with Tn5- and Tn10-derived minitransposons. Methods Enzymol. 1994;235:386-405.

3. Siehnel R, Traxler B, An DD, Parsek MR, Schaefer AL, Singh PK. A unique regulator controls the activation threshold of quorum-regulated genes in *Pseudomonas aeruginosa*. Proc Natl Acad Sci U S A. 2010;107(17):7916-21.

4. Altschul SF, Gish W, Miller W, Myers EW, Lipman DJ. Basic local alignment search tool. J Mol Biol. 1990;215(403-410).

5. Winsor GL, Griffiths EJ, Lo R, Dhillon BK, Shay JA, Brinkman Fiona SL. Enhanced annotations and features for comparing thousands of *Pseudomonas* genomes in the *Pseudomonas* genome database. Nucleic Acids Res. 2016;44(D1):D646-D53.

6. Hmelo LR, Borlee BR, Almblad H, Love ME, Randall TE, Tseng BS, et al. Precision-engineering the *Pseudomonas aeruginosa* genome with two-step allelic exchange. Nat Protoc. 2015;10(11):1820-41.

7. Byrd MS, Sadovskaya I, Vinogradov E, Lu H, Sprinkle AB, Richardson SH, et al. Genetic and biochemical analyses of the *Pseudomonas aeruginosa* Psl exopolysaccharide reveal overlapping roles for polysaccharide synthesis enzymes in Psl and LPS production. Mol Microbiol. 2009;73(4):622-38.

8. Goeres DM, Hamilton MA, Beck NA, Buckingham-Meyer K, Hilyard JD, Loetterle LR, et al. A method for growing a biofilm under low shear at the air-liquid interface using the drip flow biofilm reactor. Nat Protoc. 2009;4(5):783-8.

9. Hayden HS, Lim R, Brittnacher MJ, Sims EH, Ramage ER, Fong C, et al. Evolution of *Burkholderia pseudomallei* in recurrent melioidosis. PLoS One. 2012;7(5):e36507.

10. Li H, Durbin R. Fast and accurate short read alignment with Burrows-Wheeler transform. Bioinformatics. 2009;25(14):1754-60.

11. Chandler JR, Truong TT, Silva PM, Seyedsayamdost MR, Carr G, Radey M, et al. Bactobolin resistance is conferred by mutations in the L2 ribosomal protein. MBio. 2012;3(6).

12. Robinson JT, Thorvaldsdottir H, Winckler W, Guttman M, Lander ES, Getz G, et al. Integrative genomics viewer. Nat Biotechnol. 2011;29(1):24-6.

13. Adey A, Morrison HG, Asan, Xun X, Kitzman JO, Turner EH, et al. Rapid, low-input, low-bias construction of shotgun fragment libraries by high-density *in vitro* transposition. Genome Biol. 2010;11(12):R119.

14. McKenna A, Hanna M, Banks E, Sivachenko A, Cibulskis K, Kernytsky A, et al. The Genome Analysis Toolkit: a MapReduce framework for analyzing next-generation DNA sequencing data. Genome Res. 2010;20(9):1297-303.

15. Simon R, Priefer U, Pühler A. A broad host range mobilization system for *in vivo* genetic engineering: transposon mutagenesis in gram negative bacteria. Nat Biotechnol. 1983;1:784-91.

16. Wolter DJ, Emerson JC, McNamara S, Buccat AM, Qin X, Cochrane E, et al. *Staphylococcus aureus* small-colony variants are independently associated with worse lung disease in children with cystic fibrosis. Clin Infect Dis. 2013;57(3):384-91.

17. Smith EE, Buckley DG, Wu Z, Saenphimmachak C, Hoffman LR, D'Argenio DA, et al. Genetic adaptation by *Pseudomonas aeruginosa* to the airways of cystic fibrosis patients. Proc Natl Acad Sci U S A. 2006;103:8487-92.

18. Hickman JW, Tifrea DF, Harwood CS. A chemosensory system that regulates biofilm formation through modulation of cyclic diguanylate levels. Proc Natl Acad Sci U S A. 2005;102(40):14422-7.

19. Starkey M, Hickman J, Ma L, Zhang N, De Long S, Hinz A, et al. *Pseudomonas aeruginosa* rugose small colony variants have adaptations that likely promote persistence in the cystic fibrosis lung. J Bacteriol. 2009;191(11):3492-503.

20. Borlee BR, Goldman AD, Murakami K, Samudrala R, Wozniak DJ, Parsek MR. *Pseudomonas aeruginosa* uses a cyclic-di-GMP-regulated adhesin to reinforce the biofilm extracellular matrix. Mol Microbiol. 2010;75(4):827-42.

21. Kirisitis MJ, Prost L, Starkey M, Parsek M. Characterization of colony morphology variants isolated from *Pseudomonas aeruginosa* biofilms. Appl Environ Microbiol. 2005;71:4809-21.

22. Colvin KM, Gordon VD, Murakami K, Borlee BR, Wozniak DJ, Wong GC, et al. The Pel polysaccharide can serve a structural and protective role in the biofilm matrix of *Pseudomonas aeruginosa*. PLoS Pathog. 2011;7(1):e1001264.

23. Ma L, Jackson KD, Landry RM, Parsek MR, Wozniak DJ. Analysis of *Pseudomonas aeruginosa* conditional Psl variants reveals roles for the Psl polysaccharide in adhesion and maintaining biofilm structure postattachment. J Bacteriol. 2006;188:8213-21.

24. Zhao K, Tseng BS, Beckerman B, Jin F, Gibiansky ML, Harrison JJ, et al. Psl trails guide exploration and microcolony formation in *Pseudomonas aeruginosa* biofilms. Nature. 2013;497(7449):388-91.

25. Rual J-F, Hirozane-Kishikawa T, Hao T, Bertin N, Li S, Dricot A, et al. Human ORFeome Version 1.1: A platform for reverse proteomics. Genome Res. 2004;14:2128-35.

26. House BL, Mortimer MW, Kahn ML. New recombination methods for *Sinorhizobium meliloti* genetics. Appl Environ Microbiol. 2004;70(5):2806-15.

27. Choi K-H, Gaynor JB, White KG, Lopez C, Bosio CM, Karkhoff-Schweizer RR, et al. A Tn7-based broad-range bacterial cloning and expression system. Nat Methods. 2005;2(6):443-8.

28. Schweizer HP. *Escherichia*-*Pseudomonas* shuttle vectors derived from pUC18/19. Gene. 1991;97(1):109-12.

29. Hoang TT, Karkhoff-Schweizer RR, Kutchma AJ, Schweizer HP. A broad-host-range Flp-FRT recombination system for site-specific excision of chromosomally-located DNA sequences: application for isolation of unmarked *Pseudomonas aeruginosa* mutants. Gene. 1998;212:77-86.

30. Wolfgang MC, Lee VT, Gilmore ME, Lory S. Coordinate regulation of bacterial virulence genes by a novel adenylate cyclase-dependent signaling pathway. Dev Cell. 2003;4(2):253-63.

31. Baraquet C, Murakami K, Parsek MR, Harwood CS. The FleQ protein from *Pseudomonas aeruginosa* functions as both a repressor and an activator to control gene expression from the pel operon promoter in response to c-di-GMP. Nucleic Acids Res. 2012;40(15):7207-18.

32. Irie Y, Borlee BR, O'Connor JR, Hill PJ, Harwood CS, Wozniak DJ, et al. Self-produced exopolysaccharide is a signal that stimulates biofilm formation in *Pseudomonas aeruginosa*. Proc Natl Acad Sci U S A. 2012;109(50):20632-6.

33. Choi K-H, Schweizer HP. mini-Tn7 insertion in bacteria with single attTn7 sites: example *Pseudomonas aeruginosa*. Nat Protoc. 2006;1(1):153-61.

34. Li Z, Kosorok MR, Farrell PM, Laxova A, West SE, Green CG, et al. Longitudinal development of mucoid *Pseudomonas aeruginosa* infection and lung disease progression in children with cystic fibrosis. JAMA. 2005;293(5):581-8.
