# Supplementary material for: Elevated exopolysaccharide levels in Pseudomonas aeruginosa flagellar mutants have implications for biofilm growth and chronic infections
Source: PLoS Genet. 2020 Jun 12;16(6):e1008848. doi: 10.1371/journal.pgen.1008848 (PMC7314104; doi:10.1371/journal.pgen.1008848)
Supplement: S1 Table — (PDF) [file pgen.1008848.s002.pdf]

S1 Table. Bacterial strains.

**Strain**	**Genotype, description or relevant characteristics***	**Source**
Escherichia coli		
DH5α	Cloning strain	New England BioLabs
EC100 (λ_{pir})	Cloning strain for plasmids with R6K origin of replication	Epicenter Biotechnologies
S17.1 (λ_{pir})	Mobilization host for conjugation	[15]
SM10 (λ_{pir})	Mobilization host for conjugation	[15]
Pseudomonas aeruginosa		
AMT00071-22	clinical isolate, smooth colony morphology, clonally related to AMT00071-23	[16]
AMT00071-23	RSCV isolate from CF sputum	[16]
AMT00085-2	clinical isolate, smooth colony morphology, clonally related to AMT00085-8	[16]
AMT00085-8	RSCV isolate from CF sputum	[16]
AMT00104-8	clinical isolate, smooth colony morphology, clonally related to AMT00104-9	[16]
AMT00104-9	RSCV isolate from CF sputum, $retS_{2078C>A}$ (A693AE)	[16]
AMT00113-8	clinical isolate, smooth colony morphology, clonally related to AMT00113-6	[16]
AMT00113-6	RSCV isolate from CF sputum	[16]
AMT00115-10	clinical isolate, smooth colony morphology, clonally related to AMT00115-11	[16]
AMT00115-11	RSCV isolate from CF sputum, $wspF_{474\_477\Delta TTCGinsCAGAC}$	[16]
AMT00134-14	clinical isolate, smooth colony morphology, clonally related to AMT00134-10	[16]
AMT00134-10	RSCV isolate from CF sputum, $wspF_{635\_636\Delta CG}$	[16]
AMT00151-10	clinical isolate, smooth colony morphology, clonally related to AMT00151-11	[16]
AMT00151-11	RSCV isolate from CF sputum	[16]
AMT00159-10	clinical isolate, smooth colony morphology, clonally related to AMT00159-19	[16]
AMT00159-19	RSCV isolate from CF sputum	[16]
EX02-6A	clinical isolate, smooth colony morphology, clonally related to EX02-6B	This study
EX02-6B	RSCV isolate from explanted CF lung, $morA_{3430C>T}$ (Q1144*)	This study

11.13.7	clinical isolate, smooth colony morphology, clonally related to 11.15.3	[17]
11.15.3	RSCV isolate from CF sputum, *fleQ*$_{364C>T}$ (Q122*)	[17]
PAO1 Δ*wspR*	PAO1 with an in-frame, markerless deletion in *wspR* (derived from the Mannoil PAO1 strain passed through the laboratory of Caroline S. Harwood)	[18]
PAO1 Δ*wspR* Δ*wspF*	PAO1 Δ*wspR* with an in-frame, markerless deletion of *wspF*	[18]
PAO1$_{Parsek}$	PAO1 (derived from the Iglewski strain of PAO1 passed through the laboratory of E. Peter Greenberg)	[19]
PAO1$_{Parsek}$ Δ*pelA*	PAO1$_{Parsek}$ with a markerless deletion of *pelA* and its promoter	[20]
PAO1$_{Parsek}$ Δ*pslBDC*	PAO1$_{Parsek}$ with a markerless deletion of *pslBCD*	[21]
PAO1$_{Parsek}$ Δ*pelA* Δ*pslBDC*	PAO1$_{Parsek}$ with markerless deletions of *pelA* and its promoter as well as *pslBCD*	[20]
PAO1$_{Parsek}$ pBADpel	PAO1 with *araC* and an arabinose inducible promoter (pBAD) inserted upstream of *pelA*	[22]
PAO1$_{Parsek}$ pBADpsl	PAO1 with *araC* and an arabinose inducible promoter (pBAD) inserted upstream of *pslA*	[23]
JJH0	PAO1 wild type strain originating from the laboratory of Colin Mannoil (MPAO1), genome re-sequenced using Illumina technology	Colin Mannoil
JJH239	JJH0 *tpbB*$_{668A>G}$ (D223G), RSCV cell line isolated from a drip-flow biofilm reactor	This study
JJH251	JJH0 *wspF*$_{777C>A}$ (S259R), RSCV cell line isolated from a drip-flow biofilm reactor	This study
JJH253	JJH0 *fliM*$_{718C>T}$ (Q240*), RSCV cell line isolated from a drip-flow biofilm reactor	This study
JJH263	JJH0 *fliM*$_{915\Delta C}$ (frameshift mutant), RSCV cell line isolated from a drip-flow biofilm reactor	This study
JJH264	JJH0 *fliG*$_{128T>G}$ (V43G), RSCV cell line isolated from a drip-flow biofilm reactor	This study
JJH265	JJH0 *fliH*$_{178G>T}$ (E60*), RSCV cell line isolated from a drip-flow biofilm reactor	This study
JJH272	PAO1 Δ*wspR* Δ*fliC*	This study
JJH278	JJH0 Δ*orn*	This study
JJH282	JJH0 Δ*fliM*	This study
JJH283	JJH0 Δ*fliC*	This study
JJH286	JJH0 Δ*fliD*	This study
JJH289	JJH0 Δ*flgL*	This study
JJH294	PAO1 Δ*wspR* Δ*dsbA*	This study
JJH301	JJH0 Δ*flhA*	This study

JJH302	JJH0 Δ*dsbA*	This study
JJH305	JJH0 Δ*flgA*	This study
JJH307	JJH0 Δ*flgB*	This study
JJH309	JJH0 Δ*flgN*	This study
JJH311	$PAO1_{Parsek}$ Δ*PA5295*	This study
JJH313	$PAO1_{Parsek}$ Δ*pelA* Δ*PA5295*	This study
JJH315	$PAO1_{Parsek}$ Δ*pslBDC* Δ*PA5295*	This study
JJH317	$PAO1_{Parsek}$ Δ*pelA* Δ*pslBDC* Δ*PA5295*	This study
JJH318	$PAO1_{Parsek}$ Δ*dsbA*	This study
JJH320	$PAO1_{Parsek}$ Δ*pslBDC* Δ*dsbA*	This study
JJH321	$PAO1_{Parsek}$ Δ*pelA* Δ*pslBDC* Δ*dsbA*	This study
JJH323	$PAO1_{Parsek}$ Δ*pelA* Δ*dsbA*	This study
JJH325	$PAO1_{Parsek}$ Δ*fliC*	This study
JJH327	$PAO1_{Parsek}$ Δ*pelA* Δ*fliC*	This study
JJH329	$PAO1_{Parsek}$ Δ*pslBDC* Δ*fliC*	This study
JJH331	$PAO1_{Parsek}$ Δ*pelA* Δ*pslBDC* Δ*fliC*	This study
JJH337	PAO1 Δ*wspR* Δ*PA529* Δ*fliC*	This study
JJH339	$PAO1_{Parsek}$ Δ*orn*	This study
JJH341	$PAO1_{Parsek}$ Δ*pelA* Δ*orn*	This study
JJH343	$PAO1_{Parsek}$ Δ*pslBDC* Δ*orn*	This study
JJH345	PAO1 Δ*wspR* Δ*PA5017*	This study
JJH346	JJH0 Δ*PA5017*	This study
JJH348	$PAO1_{Parsek}$ Δ*PA5017*	This study
JJH349	$PAO1_{Parsek}$ Δ*pelA* Δ*PA5017*	This study
JJH351	$PAO1_{Parsek}$ Δ*pslBDC* Δ*PA5017*	This study
JJH353	$PAO1_{Parsek}$ Δ*pelA* Δ*pslBDC* Δ*PA5017*	This study

JJH355	$PAO1_{Parsek}$ Δ*pelA* Δ*pslBDC* Δ*orn*	This study
JJH356	JJH0 Δ*wspF*	This study
JJH357	$PAO1_{Parsek}$ Δ*wspF*	This study
JJH359	$PAO1_{Parsek}$ Δ*pelA* Δ*wspF*	This study
JJH361	$PAO1_{Parsek}$ Δ*pslBDC* Δ*wspF*	This study
JJH363	$PAO1_{Parsek}$ Δ*pelA* Δ*pslBDC* Δ*wspF*	This study
JJH367	$PAO1_{Parsek}$ Δ*retS*	This study
JJH369	$PAO1_{Parsek}$ Δ*pelA* Δ*retS*	This study
JJH371	$PAO1_{Parsek}$ Δ*pslBDC* Δ*retS*	This study
JJH373	$PAO1_{Parsek}$ Δ*pelA* Δ*pslBDC* Δ*retS*	This study
JJH377	JJH0 Δ*fliC* Δ*siaD*	This study
JJH385	JJH0 Δ*fliC* Δ*sadC*	This study
JJH393	JJH0 Δ*fliC* Δ*fleQ*	This study
JJH450	JJH0 $fliM_{718C>T}$ (Q240*)	This study
JJH451	JJH0 $fliM_{915\Delta C}$	This study
JJH452	JJH0 $fliG_{128T>G}$ (V43G)	This study
JJH453	JJH0 $fliH_{178G>T}$ (E60*)	This study
JJH454	JJH0 $wspF_{777C>A}$ (S259R)	This study
JJH455	JJH0 $tpbB_{668A>G}$ (D223G)	This study
JJH456	JJH0 *attTn7::miniTn7T*-Gm, Gm^r	This study
JJH457	JJH0 Δ*wspF* *attTn7::miniTn7T*-Gm, Gm^r	This study
JJH458	JJH0 Δ*retS* *attTn7::miniTn7T*-Gm, Gm^r	This study
JJH459	JJH0 Δ*orn* *attTn7::miniTn7T*-Gm, Gm^r	This study
JJH460	JJH0 Δ*dsbA* *attTn7::miniTn7T*-Gm, Gm^r	This study
JJH461	JJH0 Δ*fliC* *attTn7::miniTn7T*-Gm, Gm^r	This study
JJH462	JJH0 Δ*PA5017* *attTn7::miniTn7T*-Gm, Gm^r	This study

JJH463	JJH0 Δ*wspF attTn7::miniTn7T*-Gm-GW*::wspA*$_{pro}$*::wspF*, Gm^{r}	This study
JJH464	JJH0 Δ*orn attTn7::miniTn7T*-Gm-GW*::orn*, Gm^{r}	This study
JJH465	JJH0 Δ*dsbA attTn7::miniTn7T*-Gm-GW*::dsbA*, Gm^{r}	This study
JJH466	JJH0 Δ*fliC attTn7::miniTn7T*-Gm-GW*::fliC*, Gm^{r}	This study
JJH467	JJH0 Δ*PA5295 attTn7::miniTn7T*-Gm-GW*::PA5295*, Gm^{r}	This study
JJH468	JJH0 Δ*PA5295 attTn7::miniTn7T*-Gm, Gm^{r}	This study
JJH474	JJH0 Δ*PA5017 attTn7::miniTn7T*-Gm-GW*::PA5017*, Gm^{r}	This study
JJH479	JJH0 Δ*retS*	This study
JJH483	JJH0 Δ*fliC* Δ*pslD*	This study
JJH490	JJH0 Δ*fliC* Δ*pilA*	This study
JJH498	JJH0 Δ*pslD*	[24]
JJH502	JJH0 Δ*pelF* Δ*pslD*	This study
JJH504	JJH0 Δ*fliC* Δ*pelF* Δ*pslD*	This study
JJH517	JJH0 Δ*motABCD*	This study
JJH522	JJH0 Δ*fliC* Δ*pelF* Δ*pslD attTn7::miniTn7T*-Gm, Gm^{r}	This study
JJH523	JJH0 Δ*fliC* Δ*pelF* Δ*pslD attTn7::miniTn7*T2.1-Gm-GW::*pslA*$_{pro}$::*pslD-pelA*$_{pro}$::*pelF*, Gm^{r}	This study
JJH529	JJH0 Δ*pelF* Δ*pslD attTn7::miniTn7T*-Gm, Gm^{r}	This study
JJH530	JJH0 Δ*pelF* Δ*pslD attTn7::miniTn7*T2.1-Gm-GW::*pslA*$_{pro}$::*pslD-pelA*$_{pro}$::*pelF*, Gm^{r}	This study
JJH531	JJH0 Δ*retS attTn7::miniTn7T*-Gm, Gm^{r}	This study
JJH532	JJH0 Δ*retS attTn7::miniTn7T*-Gm-GW*::retS*, Gm^{r}	This study
JJH535	PAO1 Δ*wspR* Δ*orn*	This study
JJH537	PAO1 Δ*wspR* Δ*retS*	This study
JJH662	JJH0 Δ*fliC* Δ*sadB*	This study
JJH692	JJH0 Δ*fliC* Δ*fleN*	This study
JJH697	JJH0 Δ*fliC* Δ*gacS*	This study
JJH714	JJH0 Δ*fliC* Δ*PA1769*	This study

JJH720	JJH0 Δ*fliC* Δ*motAB*	This study
JJH722	JJH0 Δ*fliC* Δ*motABCD*	This study
JJH848	JJH0 $wspF_{474\_477\Delta TTCGinsCAGAC}$	This study
JJH850	JJH0 $wspF_{635\_636\Delta CG}$	This study
JJH851	JJH0 $retS_{2078C>A}$	This study
JJH853	JJH0 $morA_{3430C>T}$	This study
JJH857	JJH0 $fleQ_{364C>T}$	This study

*Gm^r, gentamicin resistance
