# Supplementary material for: Elevated exopolysaccharide levels in Pseudomonas aeruginosa flagellar mutants have implications for biofilm growth and chronic infections
Source: PLoS Genet. 2020 Jun 12;16(6):e1008848. doi: 10.1371/journal.pgen.1008848 (PMC7314104; doi:10.1371/journal.pgen.1008848)
Supplement: S2 Table — (PDF) [file pgen.1008848.s003.pdf]

**S2 Table.** Plasmids.

| Plasmid | Description or relevant characteristics* | Source |
|---|---|---|
| **Cloning, template, shuttle and helper plasmids** | | |
| pDONR221 | Entry vector for single-fragment Gateway cloning, contains donor *attP1* and *attP2* sites, Kn$^r$, Cm$^r$ | Invitrogen® |
| pDONR221 P1-P5r | Entry vector for two-fragment Multisite Gateway (GW) cloning, contains the donor *attP1* and *attP5r* sites, Kn$^r$, Cm$^r$ | Invitrogen® |
| pDONR221 P5-P2 | Entry vector for two-fragment Multisite Gateway (GW) cloning, contains the donor *attP5* and *attP2* sites, Kn$^r$, Cm$^r$ | Invitrogen® |
| pDONR223 | Entry vector for single-fragment Gateway cloning, contains donor *attP1* and *attP2*sites, Sp$^r$, Cm$^r$ | [25] |
| pMK2010 | Gateway donor plasmid, $oriT_{RP4}$, *sacB*, Kn$^r$ and Cm$^r$ | [26] |
| pTNS1 | helper plasmid for mobilizing miniTn7, Ap$^r$ | [27] |
| pUCP18 | broad host range cloning vector, Ap$^r$ | [28] |
| **Transposon mutagenesis** | | |
| pUT-miniTn5-Pro | suicide vector with miniTn5-Pro, a miniTn5 derivative containing *aac1*, an R6Kγ origin of replication, *araC* and pBAD at the O-end of the transposon, Gm$^r$, Ap$^r$ | [3] |
| **Allelic exchange vectors** | | |
| pEX18Gm | allelic exchange vector with pUC18 multiple cloning site, *sacB, oriT*, Gm$^r$ | [29] |
| pDONRPEX18Gm | pEX18Gm containing a HindIII flanked Gateway donor site from pMK2010, Gm$^r$ and Cm$^r$ | This study, [6] |
| pEX18GmGW | pEX18Gm containing the Gateway (GW) destination cloning site, Gm$^r$ | [30] |
| pEX18Gm::Δ*fleQ* | pEX18Gm containing an in-frame deletion construct for *P. aeruginosa* PAO1 *fleQ,* Gm$^r$ | [31] |
| pEX18Gm::Δ*siaD* | pEX18Gm containing an in-frame deletion construct for *P. aeruginosa* PAO1 *siaD,* Gm$^r$ | [32] |
| pEX18Gm::Δ*sadC* | pEX18Gm containing an in-frame deletion construct for *P. aeruginosa* PAO1 *sadC,* Gm$^r$ | [32] |
| pEX18Gm::Δ*pelF* | pEX18Gm containing an in-frame deletion construct for *P. aeruginosa* PAO1 *pelF*, Gm$^r$ | [22] |
| pEX19Gm::Δ*fleN* | pEX19Gm containing a nonsense allele of *fleN*; truncates the *fleN* ORF and leaves the *fliA* promoter intact | [31] |
| pHL129 | pEX18Gm containing an in-frame deletion construct for *P. aeruginosa* PAO1 *pslD*, Gm$^r$ | D. J. Wozniak |
| pJJH4 | pEX18Gm with a 549 bp in-frame deletion construct for *P. aeruginosa* PAO1 *flgN*; Gm$^r$ | This study |
| pJJH8 | pEX18Gm with a 489 bp in-frame deletion construct for *P. aeruginosa* PAO1 *flgA*, Gm$^r$ | This study |
| pJJH9 | pEX18Gm with a 472 bp in-frame deletion construct for *P. aeruginosa* PAO1 *fliM*, Gm$^r$ | This study |
| pJJH10 | pEX18Gm with a 500 bp in-frame deletion construct for *P. aeruginosa* PAO1 *fliC*, Gm$^r$ | This study |

| | | |
|---|---|---|
| pJJH11 | pEX18Gm with a 542 bp in-frame deletion construct for *P. aeruginosa* PAO1 *fliD*, $Gm^r$ | This study |
| pJJH13 | pEX18Gm with a 474 bp in-frame deletion construct for *P. aeruginosa* PAO1 *flgL*, $Gm^r$ | This study |
| pJJH14 | pEX18Gm with a 584 bp in-frame deletion construct for *P. aeruginosa* PAO1 *orn*, $Gm^r$ | This study |
| pJJH19 | pEX18Gm with a 561 bp in-frame deletion construct for *P. aeruginosa* PAO1 *flgB*, $Gm^r$ | This study |
| pJJH20 | pEX18Gm with a 668 bp in-frame deletion construct for *P. aeruginosa* PAO1 *dsbA*, $Gm^r$ | This study |
| pJJH21 | pEX18Gm with a 563 bp in-frame deletion construct for *P. aeruginosa* PAO1 *flhA*, $Gm^r$ | This study |
| pJJH24 | pEX18Gm with a 513 bp in-frame deletion construct for *P. aeruginosa* PAO1 *PA5295*, $Gm^r$ | This study |
| pJJH25 | pEX18Gm with a 694 bp in-frame deletion construct for *P. aeruginosa* PAO1 *PA5017*, $Gm^r$ | This study |
| pJJH39 | pEX18Gm with a 526 bp in-frame deletion construct for *P. aeruginosa* PAO1 *motCD,* $Gm^r$ | This study |
| pJJH43 | pEX18Gm with a 601 bp in-frame deletion construct for *P. aeruginosa* PAO1 *motAB*, $Gm^r$ | This study |
| pJJH47 | pEX18Gm with a 684 bp in-frame deletion construct for *P. aeruginosa* PAO1 *retS*, $Gm^r$ | This study |
| pJJH50 | pEX18Gm with a 879 bp deletion construct for *P. aeruginosa* PAO1 *sadB,* $Gm^r$ | This study |
| pJJH100 | pEX18GmGW containing the mutant $fliM_{718C>T}$ (Q240*) allele from *P. aeruginosa* JJH253 | This study |
| pJJH101 | pEX18GmGW containing the mutant $fliM_{915\Delta C}$ (frameshift mutation) allele from *P. aeruginosa* JJH263 | This study |
| pJJH102 | pEX18GmGW containing the mutant $fliH_{178G>T}$ (E60*) allele from *P. aeruginosa* JJH264, $Gm^r$ | This study |
| pJJH103 | pEX18GmGW containing the mutant $fliG_{128T>G}$ (V43G) allele from *P. aeruginosa* JJH265, $Gm^r$ | This study |
| PJJH104 | pEX18GmGW containing the mutant $wspF_{777C>A}$ (S259R) allele from *P. aeruginosa* JJH251, $Gm^r$ | This study |
| pJJH105 | pEX18GmGW containing the mutant $tpbB_{668A>G}$ (D223G) allele from *P. aeruginosa* JJH239, $Gm^r$ | This study |
| pJJH130 | pEX18GmGW with a 784 bp deletion construct for PAO1 *pilA*, $Gm^r$ | [24] |
| pJJH151 | pDONRPEX18Gm with a 632 bp deletion construct for *P. aeruginosa* PAO1 *PA1769*, $Gm^r$ | This study |
| pJJH230 | pDONRPEX18Gm with a 955 bp deletion construct for *P. aeruginosa* PAO1 *gacS*, $Gm^r$ | This study |
| pJJH276 | pDONRPEX18Gm containing the mutant $wspF_{474\_477\Delta TTCGinsCAGAC}$ (frameshift) allele from *P. aeruginosa* AMT00115-11 | This study |
| pJJH277 | pDONRPEX18Gm containing the mutant $wspF_{635\_636\Delta CG}$ (frameshift) allele from *P. aeruginosa* AMT00134-10 | This study |
| pJJH278 | pDONRPEX18Gm containing the mutant $retS_{2078C>A}$ (A693E) allele from *P. aeruginosa* AMT00104-09 | This study |
| pJJH282 | pDONRPEX18Gm containing the mutant $morA_{3430C>T}$ (Q1144*) allele from *P. aeruginosa* EX02-6B | This study |

| | | |
|---|---|---|
| pJJH289 | pDONRPEX18Gm containing the mutant *fleQ*$_{364C>T}$ (Q122*) allele from *P. aeruginosa* 11.15.3 | This study |
| pEX19Gm::Δ*wspF* | pEX19Gm containing an in-frame deletion allele for *P. aeruginosa* PAO1 *wspF*, Gm$^r$ | [18] |
| **Plasmids used for complementation analysis** | | |
| pUC18-miniTn7T-Gm | pUC18 containing the miniTn7 transposon, Gm$^r$, Ap$^r$ | [33] |
| pUC18-miniTn7T-Gm-GW | pUC18 containing the miniTn7 transposon with the Gateway (GW) destination cloning site, Gm$^r$, Ap$^r$, Cm$^r$ | [33] |
| pUC18-miniTn7T2-Gm-GW | pUC18-miniTn7T-Gm-GW containing synthetic tandem T4 terminators inserted at the kpnI site, Gm$^r$, Ap$^r$, Cm$^r$ | [34] |
| pJJH80 | pUC18-miniTn7T-Gm-GW with a 1510 bp fragment consisting of the *wspA* promoter region (483 bp) fused to the *wspF* ORF, Gm$^r$, Ap$^r$ | This study |
| pJJH82 | pUC18-miniTn7T-Gm-GW with a 3075 bp fragment encoding *retS* and its native promoter, Gm$^r$, Ap$^r$ | This study |
| pJJH83 | pUC18-miniTn7T-Gm-GW with a 747 bp fragment encoding *orn* and its native promoter, Gm$^r$, Ap$^r$ | This study |
| pJJH84 | pUC18-miniTn7T-Gm-GW with a 849 bp fragment encoding *dsbA* and its native promoter, Gm$^r$, Ap$^r$ | This study |
| pJJH85 | pUC18-miniTn7T-Gm-GW with a 1852 bp fragment encoding *fliC* and its native promoter, Gm$^r$, Ap$^r$ | This study |
| pJJH86 | pUC18-miniTn7T-Gm-GW with a 1955 bp fragment encoding *PA5295* and its native promoter, Gm$^r$, Ap$^r$ | This study |
| pJJH124 | pUC18-miniTn7T-Gm-GW with a 3182 bp fragment encoding *PA5017* and its native promoter, Gm$^r$, Ap$^r$ | This study |
| pJJH148 | pUC18-miniTn7T2-Gm-GW wiith a 644 bp fragment encoding *flgB* and its native promoter, Gm$^r$, Ap$^r$ | This study |
| pJJH149 | pUC18-miniTn7T2-Gm-GW containing two divergently transcribed operons: 1) a 1369 bp DNA fragment encoding the *pslA* promoter fused to the *pslD* ORF, and 2) a 2062 bp DNA fragment encoding the *pelA* promoter fused to the *pelF* ORF, Gm$^r$, Ap$^r$ | This study |
| pUCP18*::wspF* | pUCP18 containing *P. aeruginosa* PAO1 *wspF*, Ap$^r$ | [19] |

*Ap$^r$, ampicillin resistance; Cm$^r$, chloramphenicol resistance; Gm$^r$, gentamicin resistance; Sp$^r$, spectinomycin resistance.
