# Supplementary material for: Elevated exopolysaccharide levels in Pseudomonas aeruginosa flagellar mutants have implications for biofilm growth and chronic infections
Source: PLoS Genet. 2020 Jun 12;16(6):e1008848. doi: 10.1371/journal.pgen.1008848 (PMC7314104; doi:10.1371/journal.pgen.1008848)
Supplement: S3 Table — (PDF) [file pgen.1008848.s004.pdf]

S3 Table. Primers and sequencing adapters.

Oligonucleotide	DNA sequence#
**DNA Sequencing and PCR detection of mutant alleles**	
oJJH1072_fliGF02-SEQ	GTC GTG AAA GAG TGG ATC AAC
oJJH1073_fliGR02-SEQ	GGA TTG TCT TTG TCC TTG TCG
oJJH1074_fliGF03-SEQ	GCC AAA CTG ACC AAG GTC G
oJJH517_fliHSEQF01	CCA CGA CAA GGA CAA AG
oJJH518_fliHSEQR01	CAA GGC GCA TCG ACG TC
oJJH1069_fliMF03-SEQ	GGC CAA CTG GCT ATC GAA C
oJJH1071_fliMF04-SEQ	GAC GCC AAG CTG GTG TTC
oJJH514_fliMSEQF01	CAG GAT GAA ATC GAC GCG
oJJH367_M13-Universal-F	GTA AAA CGA CGG CCA G
oJJH368_M13-Universal-R	CAG GAA ACA GCT ATG AC
para2	CAT TGA TTA TTT GCA CGG CGT
oJJH373_PglmS-down	GCA CAT CGG CGA CGT GCT CTC
oJJH374_PglmS-up	CTG TGC GAC TGC TGG AGC TGA
oJJH369_Gm-up	TGG AGC AGC AAC GAT GTT AC
oJJH370_Gm-down	TGT TAG GTG GCG GTA CTT GG
oJJH507_Tn7LF01	GTG TAA AGC CTG GGG TGC
oJJH519_tpbBSEQF01	CAA CCA GCA ACT GAT CG
oJJH515_wspFSEQF01	GTC AAT GAC ATG CCG TTG
oJJH516_wspFSEQR01	TCG AAT ACC TCC GCC AG
oJJH1077_fleQF01-SEQ	GAT CAG GTC TGT CAG GTC AAG
oJJH1077_fleQR01-SEQ	CAG AGC ATG CCC ACC AAC G
oJJH1367_sadCF02-SEQ	GTT GAG CAT GGC CAT CTT CG
oJJH1368_sadCR02-SEQ	CAG CCA TGG GTC CTC GTG
oJJH1371_sadBF02-SEQ	CCA CTG CGT GCT GCT CTA C
oJJH1372_sadBR02-SEQ	CAG CTC GAG GGC AAG GAC
oJJH1398_siaDF01-SEQ	GTG ATC GAC TGG GTC GAG C
oJJH1398_siaDR01-SEQ	CGA GGG ACA GGA CAT GGA C
**Construction of allelic exchange vectors (restriction cloning)**	
oJJH106_dsbAupF1	ATC CGG **AAG CTT** CCT ACG TGG CCA AGC AGC
oJJH107_dsbAupR1	*CCT TGC CTA CTT CTT GGC CGC TGC* GAG AAT CAG GTT ACG CAT CG
oJJH108_dsbAdownF1	GCA GCG GCC AAG AAG TAG
oJJH109_dsbAdownR1	ATC CGG **GAA TTC** CCT GGT TGA CGT AGC CGG
oJJH138_flgAupF1	ATC CGG **AAG CTT** CAC GTT GAT GCC GTA GAG C
oJJH139_flgAupR1	*GCT TTC CCT CTA CAT ATT CAC CTC GAC* GCC GAG CCC GAG CAA AAC
oJJH140_flgAdownF1	GTC GAG GTG AAT ATG TAG AGG

oJJH141_flgAdownR1	ATC CGG **GAA TTC** CGG TGC TGC CGG TAC G
oJJH142_flgBupF1	ATC CGG **AAG CTT** CAC GAG TGA GCC GTG TC
oJJH143_flgBupR1	*CAT GGC GGT TAC TCT CCA CGC A* **G** G TCG AAA CTG ATG CTC ATG G
oJJH144_flgBdownF1	CTG CGT GGA GAG TAA CCG
oJJH145_flgBdownR1	ATC CGG **GAA TTC** GCA TCA GCG AAC TCT GGT C
oJJH86_flgLupF1	ATC CGG **AAG CTT** GCC TCG CAG ACC GTG CTC
oJJH87_flgLupR1	*CGC TTA ACT CAG GTA GTT GAA CAG* CTG GAT CGT TGA AAT GCG CAT
oJJH88_flgLdownF1	CTG TTC AAC TAC CTG AGT TAA GC
oJJH89_flgLdownR1	ATC CGG **CTG CAG** GCG ACG CGC AGT TTC TCG
oJJH134_flgNupF1	ATC CGG **AAG CTT** CGA GTC GGT GCA GAT CAG
oJJH135_flgNupR1	*GGT TCA GGC CTG GCT GAG CGG* GGT AGG GGA GTC AGG CAT
oJJH136_flgNdownF1	CCG CTC AGC CAG GCC
oJJH137_flgNdownR1	ATC CGG **GAA TTC** GGA TGA GTT CGT CCA GCG
oJJH122_flhAupF1	ATC CGG **AAG CTT** GCG ATT ACG CTA GCT CAT CG
oJJH123_flhAupR1	*CGT TCA GTT CTG TCC CAC CGT CGC* CAG TTG CGT GCG ATC CAC
oJJH124_flhAdownF1	GCG ACG GTG GGA CAG AAC
oJJH125_flhAdownR1	ATC CGG **GAA TTC** GCT CCA CTT CCA GCG CC
oJJH80_fliCupF1	ATC CGG **AAG CTT** CCT CTG CCG GAC CAA CC
oJJH81_fliCupR1	*GGC TTA GCG CAG CAG GCT CAG* CGT GTT GAC TGT AAG GGC CAT
oJJH82_fliCdownF1	CTG AGC CTG CTG CGC TAA G
oJJH83_fliCdownR1	ATC CGG **CTG CAG** CGC GAG ATT CGC TGG CC
oJJH92_fliDupF1	ATC CGG **AAG CTT** GCA ACT TGA ACT TCA GCA TCG
oJJH93_fliDupR1	*GAA TCA GGT CTT CTT GCC GCT GCC* TCC TAT CGA GAT ACC GGC CAT
oJJH94_fliDdownF1	GGC AGC GGC AAG AAG ACC
oJJH95_fliDdownR1	ATC CGG **CTG CAG** CAC GCG CCT GGG CCA G
oJJH74_fliMupF1	ATC CGG **AAG CTT** GGC GCA GAT GGA CGC ATT G
oJJH75_fliMupR1	*CGG TCA GCG CGA GCG CTC GAC* CAG ATC TTG CAC GGC CAT G
oJJH76_fliMdownF1	GTC GAG CGC TCG CGC TGA
oJJH77_fliMdownrR1	ATC CGG **GAA TTC** GGC GAA GCG CCG AAC TC
oJJH223_motAupF1	ATC CGG **AAG CTT** CCT GCA ACT GGT CGG C
oJJH235_DOUBLEmotABupR1	*GAA CCT CAG TCG CCC TTG ATC TG* C TCG ATG CCG ATG ATT TTT GAC
oJJH229_motBdownF1	GAG CAG ATC AAG GGC GAC
oJJH230_motBdownR1	ATC CGG **GAA TTC** CAC CGC GCG ACT GTT C
oJJH214_motCupF1	ATC CGG **AAG CTT** CGC AGG ACG AGG CCA G
oJJH222_DOUBLEmotCDupR1	*CAT GGC GAA GGC GAC GGG GAA TTG AC* G ACC AGG CTG AGC ACA TC
oJJH220_motDdownF1	GTC AAT TCC CCG TCG CC
oJJH221_motDdownR1	ATC CGG **GAA TTC** GCT GTG CTC GAG GGT G
oJJH98_ornupF1	ATC CGG **AAG CTT** GAG CGA TCA CCT GGC CG

oJJH99_ornupR1	*GGA TCA GAG CTT GAT GAA GTG* GAT AAG GTT CTG CGG GTT CTG
oJJH100_orndownF1	GAC CAC TTC ATC AAG CTC TGA TC
oJJH101_orndownR1	ATC CGG **CTG CAG** GGA CAA CGC CGA ACT CGC
oJJH236_PA5017upF1	ATC CGG **AAG CTT** CGA GCA TGC CAC GCT C
oJJH237_PA5017upR1	*GTG CAG GGT GCG GCA GGG ATA GCG* GGC ATC GGG ATG ACT TTT C
oJJH238_PA5017downF1	CGC TAT CCC TGC CGC
oJJH239_PA5017downR1	ATC CGG **GAA TTC** AGC AGC CCA TGC CGA AC
oJJH189_PA5295upF2	ATC CGG **AAG CTT** CAC GCT GGT CTT GCC G
oJJH190_PA5295upR2	*ACC GAA ATC GCG CAA CTG CTC CTG* GGG CGC TGA CAA GCA AC
oJJH191_PA5295downF2	CAG GAG CAG TTG CGC G
oJJH192_PA5295downR2	ATC CGG **GAA TTC** GCC ATT GGC CTG ACC C
oJJH240_retSupF1	ATC CGG **AAG CTT** GGT CAT CGA AGG CCT GG
oJJH241_retSupR1	*CAG TAG GCG ATC AGC TCA CGC AAT TG*G AGT AGT CCT ATG GCG ATC
oJJH242_retSdownF1	CAA TTG CGT GAG CTG ATC G
oJJH243_retSdownR1	ATC CGG **GAA TTC** GTG CGG TTA TCC GCC G
oJJH252_sadBupF2	ATC CGG **AAG CTT** TCC TGG CGC ACT GGC C
oJJH253_sadBupR2	GTC ACC CCG GCA AGC GAT TGA ACT GGA GCA GCT GGA GGA TGA C
oJJH254_sadBdownF2	CAG TTC AAT CGC TTG CCG
oJJH255_sadBdownR2	ATC CGG **GAA TTC** GCC ACT ACC TGG TCA GG

Construction of allelic exchange vectors (via Gateway cloning)

oJJH469_attP1-GWcassette201-F02	ATC CGG **AAG CTT** GTT AAC GCT AGC ATG GAT CTC
oJJH470_attP2-GWcassette201-R02	ATC CGG **AAG CTT** TCA TGA TGA TAT ATT TTT ATC TTG TGC
oJJH436_fliGF01-GWB1	**GGG GAC AAG TTT GTA CAA AAA AGC AGG CTC A**AT GAG TGA GAA TCG TCT CGC
oJJH437_fliGR01-GWB2	**GGG GAC CAC TTT GTA CAA GAA AGC TGG GTG** TCA GAT CAT CTC CTC GCC A
oJJH438_fliHF01-GWB1	**GGG GAC AAG TTT GTA CAA AAA AGC AGG CTC A**GT GGT CCC CCA CGA CAA
oJJH439_fliHR01-GWB2	**GGG GAC CAC TTT GTA CAA GAA AGC TGG GTG** TCA AGG CGC ATC GAC GTC
oJJH434_fliMF01-GWB1	**GGG GAC AAG TTT GTA CAA AAA AGC AGG CTC A**AT GGC CGT GCA AGA TCT G
oJJH435_fliMR01-GWB2	**GGG GAC CAC TTT GTA CAA GAA AGC TGG GTG** TCA GCG CGA GCG CTC G
oJJH1302_morAF01-GWB1	**GGG GAC AAG TTT GTA CAA AAA AGC AGG CTC A**GT GTC GAC CCC CTC GCT AAC
oJJH1303_morAR01-GWB2	**GGG GAC CAC TTT GTA CAA GAA AGC TGG GTA** TCA GCC CTC GTT GAA CAT GAA C
oJJH1159_retSF02-GWB1	**GGG GAC AAG TTT GTA CAA AAA AGC AGG CTC** AGT GGT ACG GCT TCG GAT CG
oJJH1160_retSR02-GWB2	**GGG GAC CAC TTT GTA CAA GAA AGC TGG GTA** CAC GTC GCT GCC CTC AGG
oJJH442_tpbBF01-GWB1	**GGG GAC AAG TTT GTA CAA AAA AGC AGG CTC A**AT GAA CCG TCG TCG TCG C
oJJH443_tpbBR01-GWB2	**GGG GAC CAC TTT GTA CAA GAA AGC TGG GTG** CTA CTT GGG TGG AGC CTC
oJJH440_wspFF01-GWB1	**GGG GAC AAG TTT GTA CAA AAA AGC AGG CTC A**TT GAG GAT CGG AAT CGT CAA T
oJJH441_wspFR01-GWB2	**GGG GAC CAC TTT GTA CAA GAA AGC TGG GTG** CTA ATC GAA TAC CTC CGC CA
oJJH520_PA1769upF1-GWB1	**GGG GAC AAG TTT GTA CAA AAA AGC AGG CTC A**CG GTT GTG GCA AAT CCG
oJJH409_PA1769upR1	*GAG CGG TTA TTT GAA GCG CCG TTC* GGT TCG TTT CAT TTG CAC C

oJJH410_PA1769downF1	GAA CGG CGC TTC AAA TAA C
oJJH521_PA1769downR1-GWB2	**GGG GAC CAC TTT GTA CAA GAA AGC TGG GTG** GTC CTG GTT GGT GTC GC
oJJH1095_gacSupF01-GWB1	**GGG GAC AAG TTT GTA CAA AAA AGC AGG CTC** GCT GAC ATC AGG ATC ACC G
oJJH1096_gacSupR01	*GCA TGG TCA GAG TTC GCT GGA GTC GAG* CCC CTT GAT GCC GAG ATC C
oJJH1097_gacSdownF01	CTC GAC TCC AGC GAA CTC TG
oJJH1098_gacSupR01-GWB2	**GGG GAC CAC TTT GTA CAA GAA AGC TGG GTA** GTC CGG TGC CGA TCA CTC

Construction of pUC18-miniTn7T-Gm-GW vectors (Gateway cloning)

oJJH359_dsbAF01-GWB1	**GGG GAC AAG TTT GTA CAA AAA AGC AGG CT**C AGC TAC ATC CAG GGT CT
oJJH360_dsbAR01-GWB2	**GGG GAC CAC TTT GTA CAA GAA AGC TGG GT**C CGA ATC ACC TTG CCT AC
oJJH508_flgBF01-GWB1	**GGG GAC AAG TTT GTA CAA AAA AGC AGG CTC G**CC GGA AGC ATG GGA AAG G
oJJH509_flgBR01-GWB2	**GGG GAC CAC TTT GTA CAA GAA AGC TGG GTA** TTA CTC TCC ACG CAG GGC
oJJH363_fliCF01-GWB1	**GGG GAC AAG TTT GTA CAA AAA AGC AGG CT**A ATC CGG GTT TTT CTC GAA C
oJJH364_fliCR01-GWB2	**GGG GAC CAC TTT GTA CAA GAA AGC TGG GT**C GCG TGA GTG ACC GTT C
oJJH361_ornF01-GWB1	**GGG GAC AAG TTT GTA CAA AAA AGC AGG CT**T GGC GTT TGG CCA TGA TG
oJJH362_ornR01-GWB2	**GGG GAC CAC TTT GTA CAA GAA AGC TGG GT**C GCG AGC ACG TGG AAT G
oJJH432_PA5017F02-GWB1	**GGG GAC AAG TTT GTA CAA AAA AGC AGG CTC A**AG CTG CTG GCG GAC ATC
oJJH433_PA5017R02-GWB2	**GGG GAC CAC TTT GTA CAA GAA AGC TGG GTG** CGA TCA GTG CAG GGT GC
oJJH357_PA5295F01-GWB1	**GGG GAC AAG TTT GTA CAA AAA AGC AGG CT**C GGA AGA GAG GGT TGA AG
oJJH358_PA5295R01-GWB2	**GGG GAC CAC TTT GTA CAA GAA AGC TGG GT**G CCT TCG AGT GGA AGA CC
oJJH372_PTn7L	ATT AGC TTA CGA CGC TAC ACC C
oJJH371_PTn7R	CAC AGC ATA ACT GGA CTG ATT TC
oJJH365_retSF01-GWB1	**GGG GAC AAG TTT GTA CAA AAA AGC AGG CT**G AGA AGA TCC GCT GGA AC
oJJH366_retSR01-GWB2	**GGG GAC CAC TTT GTA CAA GAA AGC TGG GT**AG CCG CGT GCG GTT ATC
oJJH385_wspAProF01-GWB1	**GGG GAC AAG TTT GTA CAA AAA AGC AGG CT**G TTC CCG ACC CGC ATC
oJJH386_wspA(pro)R01	*GTC ATT GAC GAT TCC GAT CCT CAA* ATT TTT CCC CAA GAC TCG AGT
oJJH387_wspFF01	TTG AGG ATC GGA ATC GTC AAT
oJJH388_wspFR01-GWB2	**GGG GAC CAC TTT GTA CAA GAA AGC TGG GT**T GGC CGG AGT CGA ACC

Construction of pUC18-miniTn7T2-Gm-GW vectors (Multisite Gateway cloning)

oJJH498_pslAProR01-GWB5r	*TGA TGT TCA CTT CCA GTA GCC TGG* CGA CTT CGA ATG CAT GTT GTT TG
oJJH499_pslAProR01	*TGA TGT TCA CTT CCA GTA GCC TGG* CGA CTT CGA ATG CAT GTT GTT TG
oJJH500_pslDF01	CCA GGC TAC TGG AAG TGA AC
oJJH501_pslDR01-GWB1	**GGG GAC AAG TTT GTA CAA AAA AGC AGG CTA A**TC AGC GAT CAT TGT TGA CGG
oJJH502_pelAProF01-GWB5	**GGG GAC AAC TTT GTA TAC AAA AGT TGT C**GT TCC TCG CAC GCA ACT G
oJJH503_pelAProR01	*GTT CGG TCA TGT CCA GTA TCT CGC* GAT TCC TTT CTT GCT GAA CCG
oJJH504_pelFF01	GCG AGA TAC TGG ACA TGA CC
oJJH505_pelFR01-GWB2	**GGG GAC CAC TTT GTA CAA GAA AGC TGG GTA** TCA TGC AAT CTC CGT GGC TT

Adaptors used for multiplexed genome sequencing

PE_Adapt_hi	/5Phos/GAT CGG AAG AGC GGT TCA GCA GGA ATG CCG AG

PE_Adapt_lo_1	AC ACT CTT TCC CTA CAC TAC GAA GTC ATC *T
PE_Adapt_lo_2	AC ACT CTT TCC CTA CAC AGG ACA GTT ATC *T
PE_Adapt_lo_3	AC ACT CTT TCC CTA CAC GTC ATG CAT ATC *T
PE_Adapt_lo_4	AC ACT CTT TCC CTA CAC CTC AGA TCA ATC *T
PE_Adapt_lo_5	AC ACT CTT TCC CTA CAC TCG TTG AAG ATC *T
PE_Adapt_lo_6	AC ACT CTT TCC CTA CAC CAT TAA CGC ATC *T
PE_Adapt_lo_7	AC ACT CTT TCC CTA CAC ACC GTA AGA ATC *T
PE_Adapt_lo_8	AC ACT CTT TCC CTA CAC GAC GAG ATT ATC *T

#*Italics* denote a region of complementarity for SOE-PCR; underlined sequence denotes a region specific to the targeted DNA amplicon; **bold** denotes an attachment (*att*) or restriction site for cloning; /5Phos/ denotes 5’-phosphorylation of the oligonucleotide; an asterisk “*” indicates a phosphorothiolate bond modification
