# Supplementary figures and images for: Elevated exopolysaccharide levels in Pseudomonas aeruginosa flagellar mutants have implications for biofilm growth and chronic infections
Source: PLoS Genet. 2020 Jun 12;16(6):e1008848. doi: 10.1371/journal.pgen.1008848 (PMC7314104; doi:10.1371/journal.pgen.1008848)

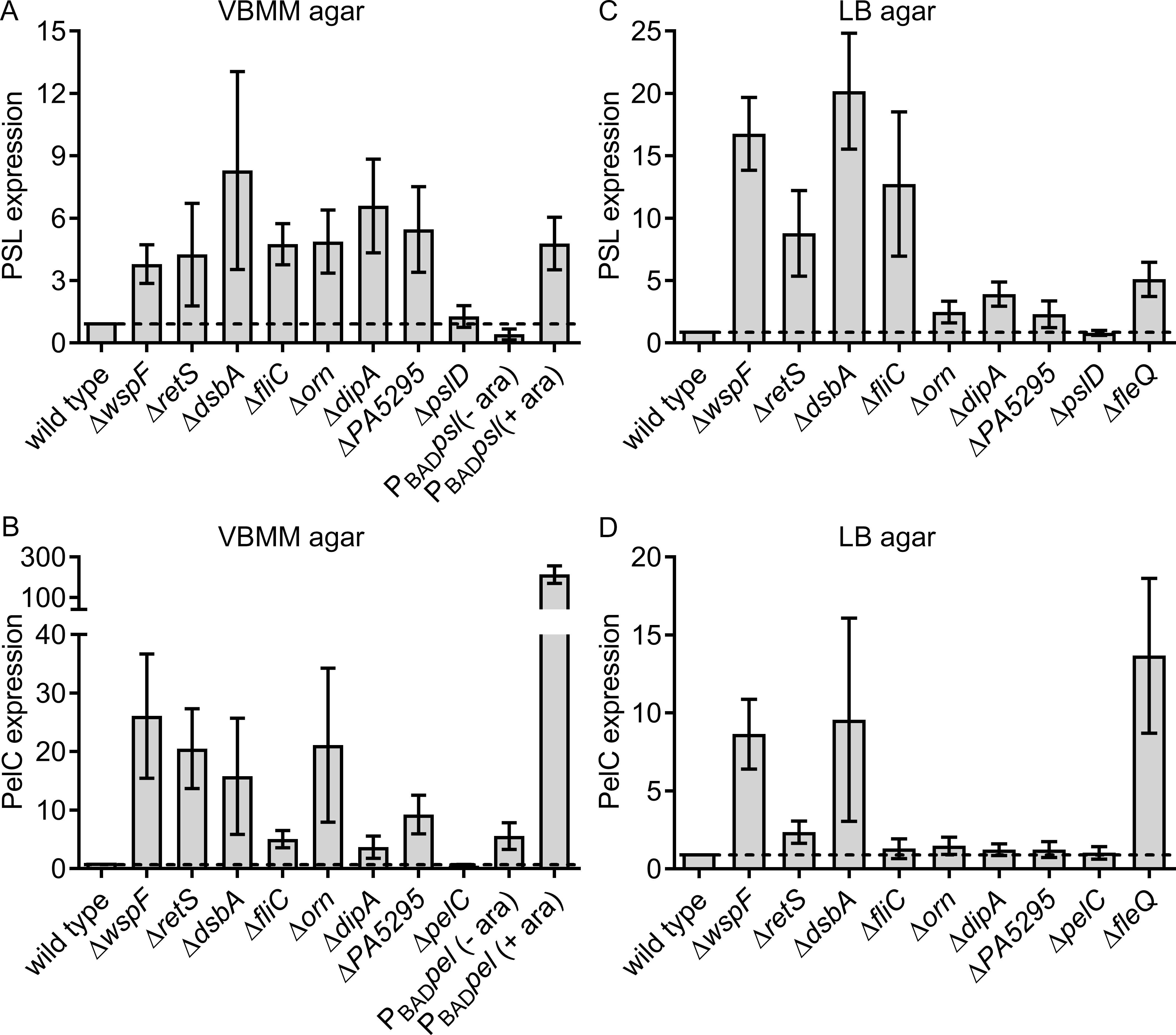

Supplement: S1 Fig — (A) Semi-quantitative dot blots for the Psl polysaccharide from strains grown on VBMM agar. (B) Semi-quantitative Western blots for PelC from strains grown on VBMM agar. (C) Semi-quantitative dot blots for the Psl polysaccharide from strains grown on LB agar. (D) Semi-quantitative Western blots for PelC from strains grown on LB agar. Each bar indicates the mean and SD for 1 or 2 technical replicates from each of 3 to 4 independent biological replicates. (TIF) [file pgen.1008848.s005.tif]

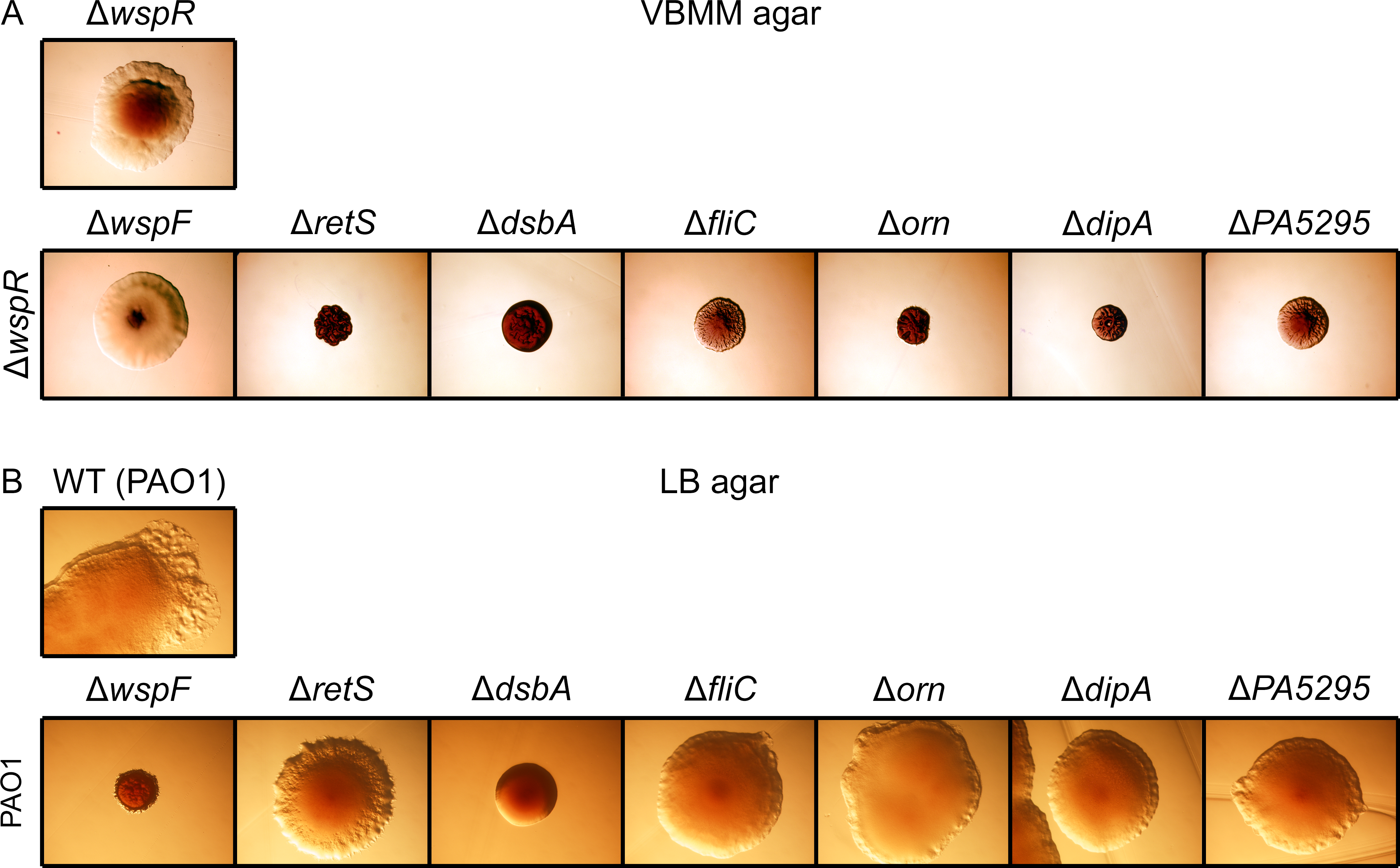

Supplement: S2 Fig — (A) The diguanylate cyclase WspR is dispensable for the nutrition-dependent RSCV phenotype resulting from RSCV-linked mutations identified in this study. Bacteria in these panels were cultured and photographed on VBMM agar containing Congo red and brilliant blue R (see Material and Methods). (B) These nutrition-dependent RSCV-linked genotypes give rise to bacterial colonies with smooth morphology on LB agar. LB agar was also supplemented with the dyes Congo red and brilliant blue R. Each panel represents an area that is approximately 5.0 × 3.5 mm. (TIF) [file pgen.1008848.s006.tif]

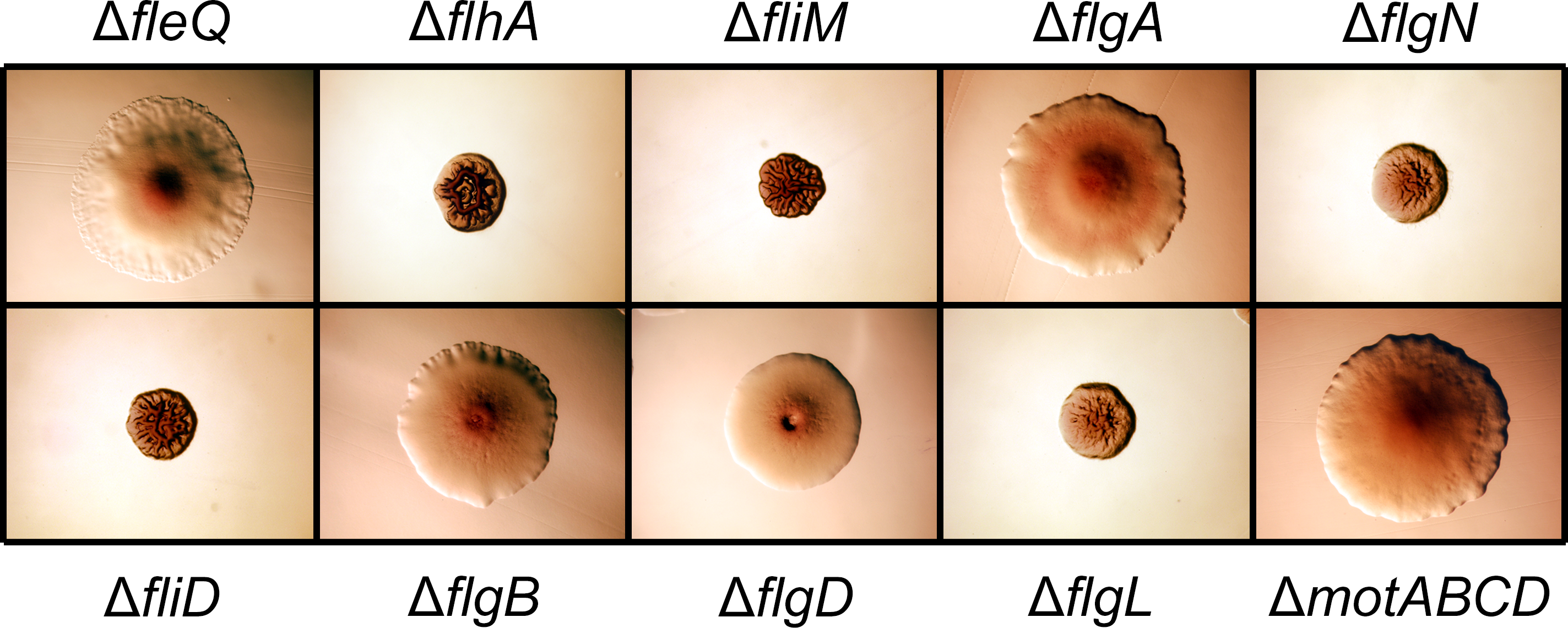

Supplement: S3 Fig — Precisely defined in-frame deletion mutations were introduced into a series of flagellar genes in wild type PAO1 strain. Only some of these mutations caused the RSCV phenotype. In all panels, bacteria were cultured and photographed on VBMM agar containing Congo red and brilliant blue R (see Material and Methods). Each panel represents an area that is approximately 5.0 mm × 3.5 mm. (TIF) [file pgen.1008848.s007.tif]

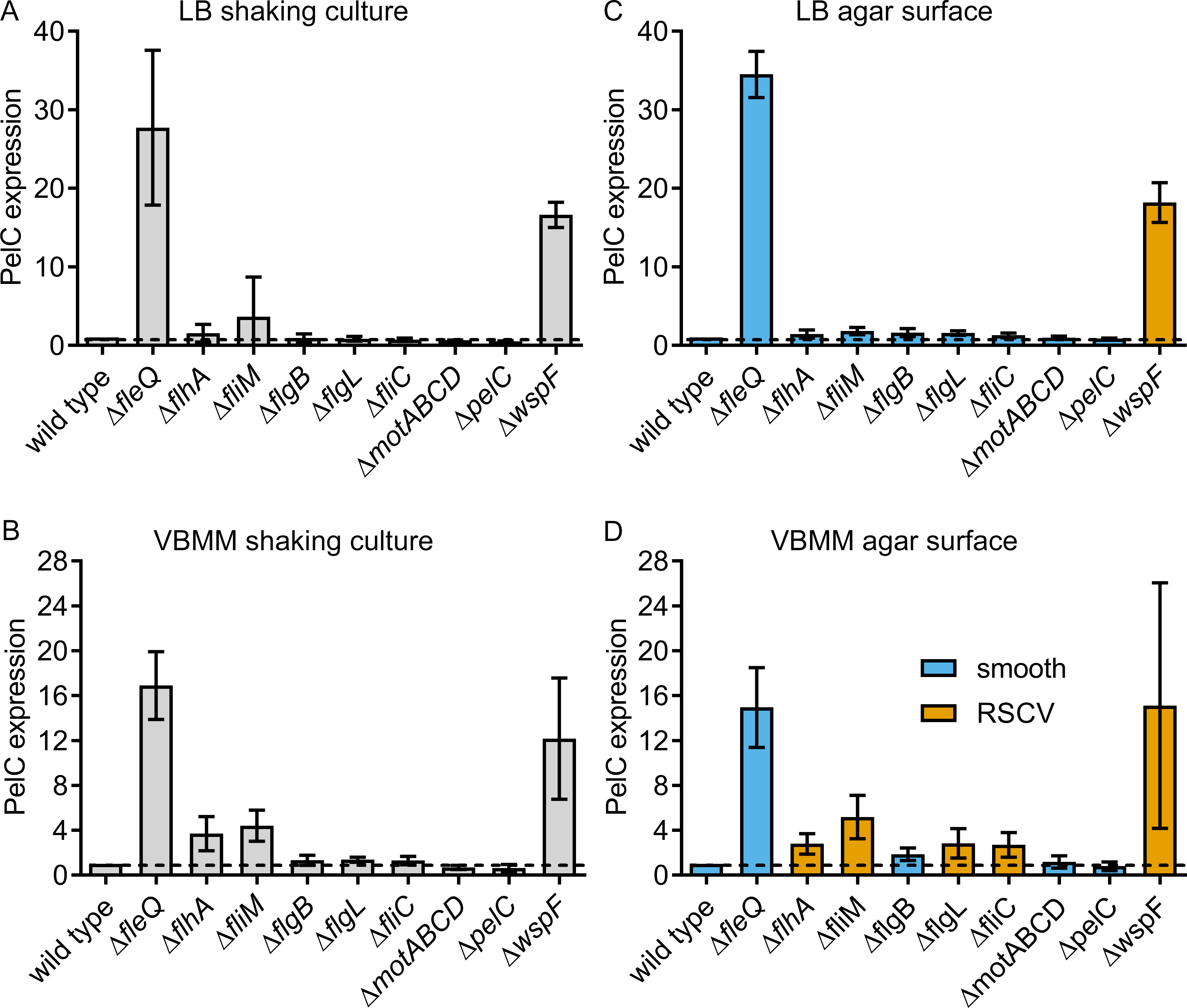

Supplement: S4 Fig — Semi-quantitative Western blots for PelC from flagellar mutants grown (A and B) in shaken LB or VBMM cultures, or (C and D) on the surface of LB or VBMM agar, respectively. Each bar indicates the mean and standard deviation for 1 or 2 technical replicates from each of 3 to 4 independent biological replicates. (TIF) [file pgen.1008848.s008.tif]

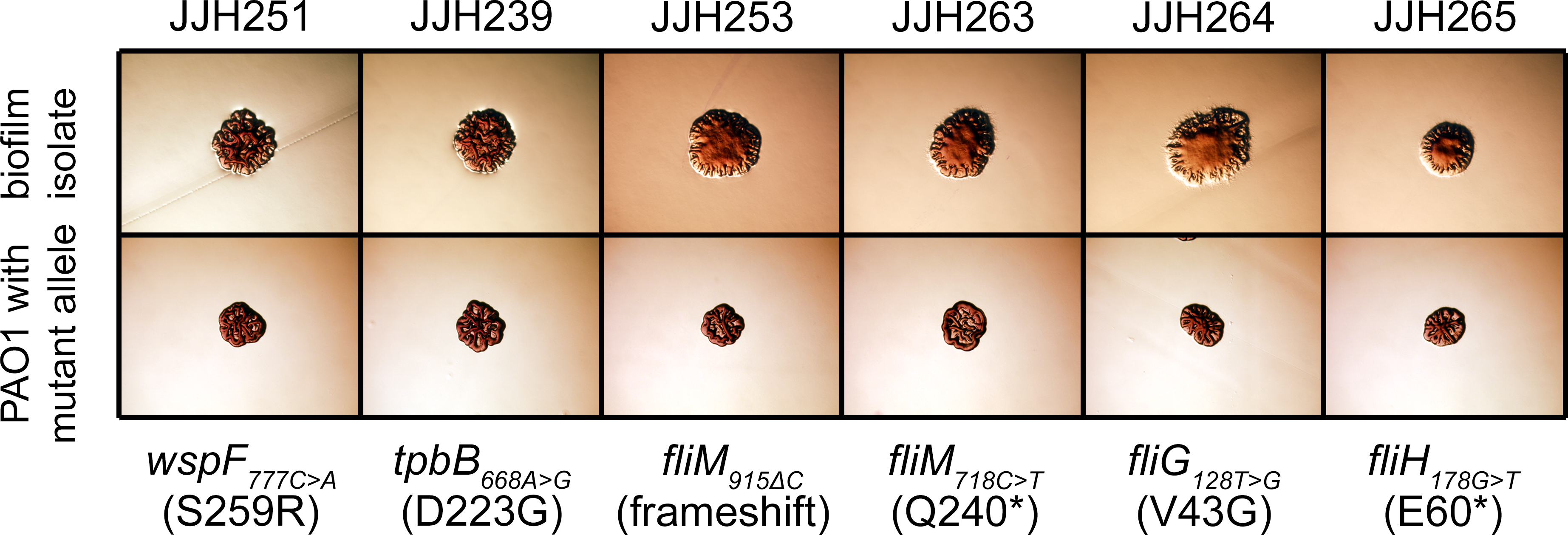

Supplement: S5 Fig — Photographs of agar-grown RSCVs that were isolated from biofilm reactors after experimental evolution. RSCV-linked mutations were identified by whole genome sequencing (top). Colony morphology of mutants in which the RSCV-linked allele from the biofilm isolate was introduced into the ancestral PAO1 strain (bottom). In all panels, bacteria were cultured and photographed on VBMM agar containing Congo red and brilliant blue R (see Material and Methods). Each panel represents an area that is approximately 5.0 mm × 3.5 mm. (TIF) [file pgen.1008848.s009.tif]

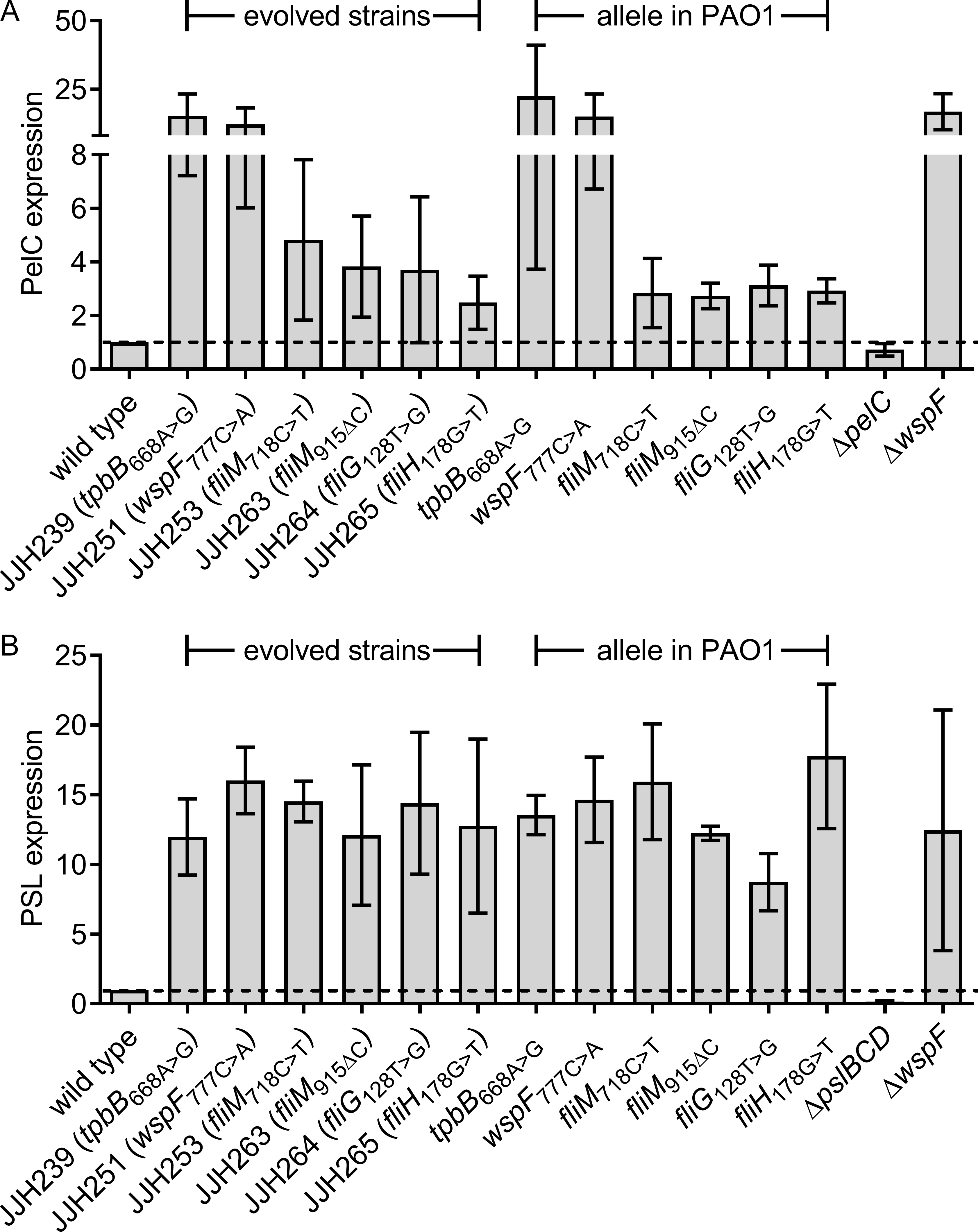

Supplement: S6 Fig — (A) Semi-quantitative PelC Western blots for strains grown on VBMM agar. (B) Semi-quantitative Psl dot blots for strains grown on VBMM agar. Each bar indicates the mean and standard deviation for 3 to 6 independent biological replicates. (TIF) [file pgen.1008848.s010.tif]

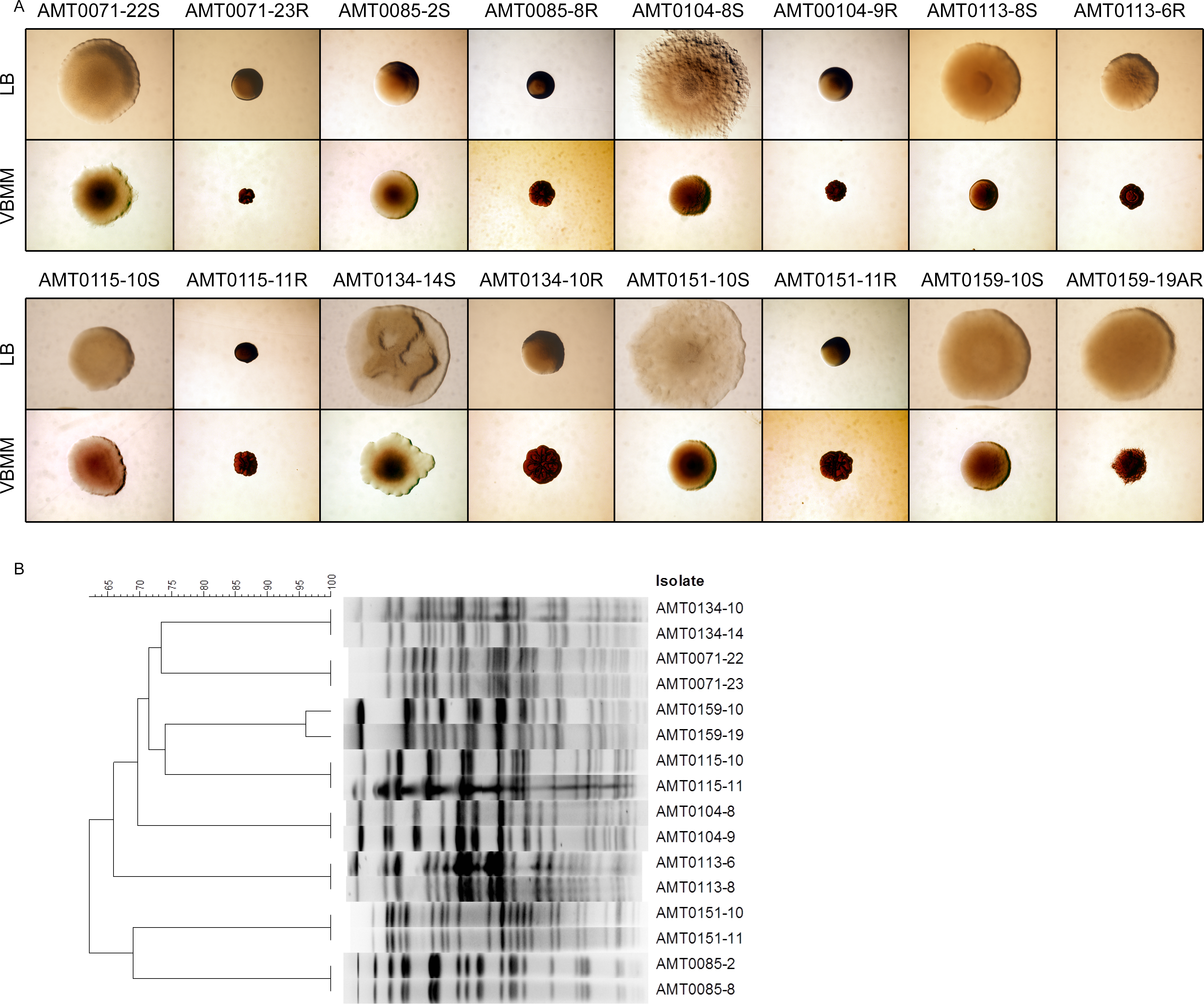

Supplement: S7 Fig — (A) Colony morphology of isolates on LB and VBMM agar. Each panel represents an area that is approximately 5.0 mm × 3.5 mm. (B) Analysis of pulsed-field gel electrophoresis (PFGE) data indicating that pairs of CF isolates with smooth and RSCV colony phenotypes are close genetic relatives. Phylogenetic relationships were calculated from existing PFGE data for the PASA collection of P. aeruginosa isolates at Seattle Children’s Hospital [40] using Bionumerics Seven (Applied Maths). (TIF) [file pgen.1008848.s011.tif]

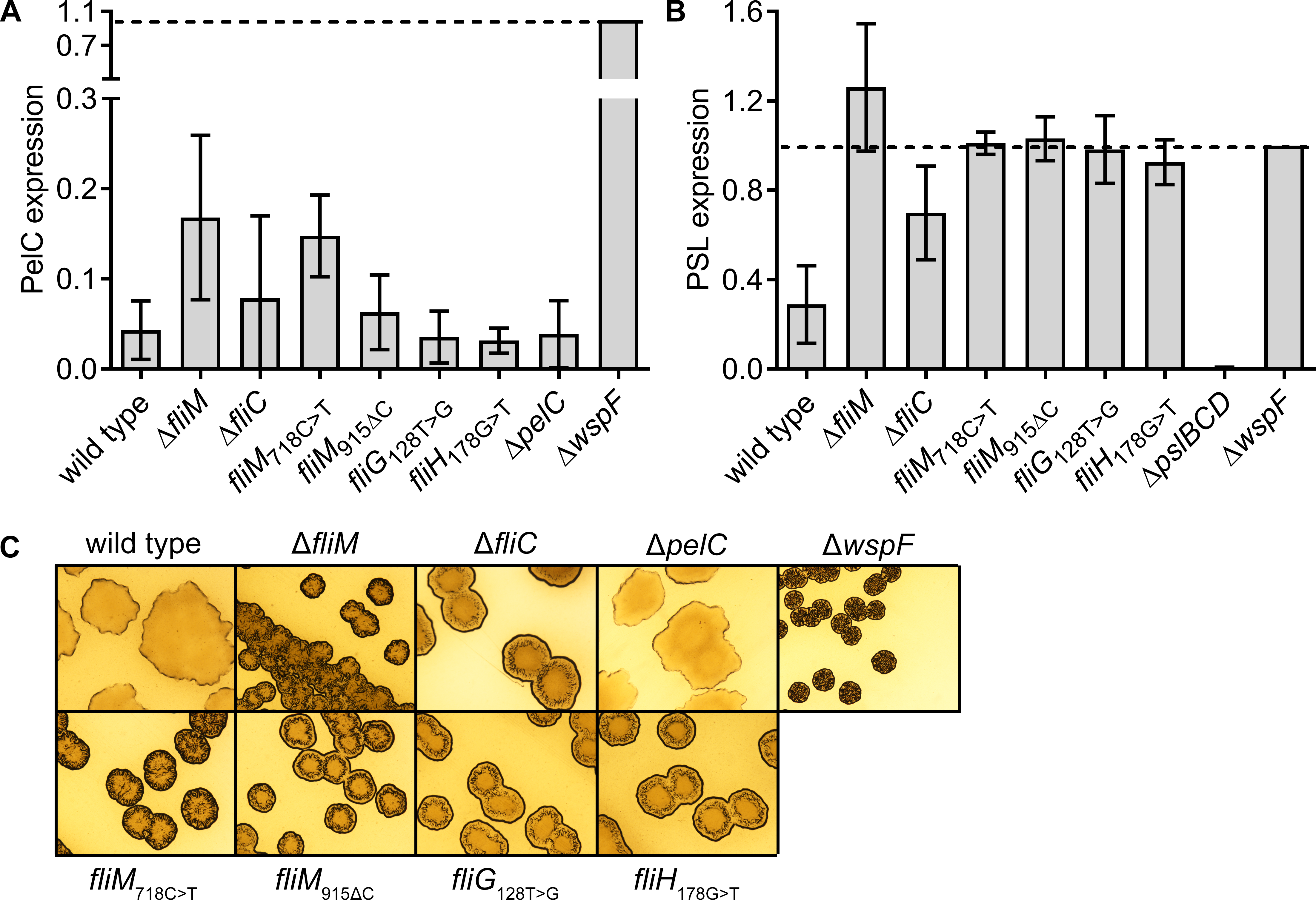

Supplement: S8 Fig — (A) Semi-quantitative Western blots for PelC from strains grown on SCFM agar. (B) Semi-quantitative dot blots for the Psl polysaccharide from strains grown on SCFM agar. (C) Colony morphology of flagellum mutants on SCFM agar. Each panel represents an area that is approximately 5.0 mm × 3.5 mm. Each bar indicates the mean and SD for 3 biological replicates. Relative expression levels have been normalized to the ΔwspF strain. (TIF) [file pgen.1008848.s012.tif]
